# Endosymbionts modulate virus effects on aphid-plant interactions

**DOI:** 10.1038/s41396-023-01549-z

**Published:** 2023-11-18

**Authors:** Patricia Sanches, Consuelo M. De Moraes, Mark C. Mescher

**Affiliations:** Department of Environmental Systems Science, ETH Zürich, Zürich, Switzerland

**Keywords:** Microbial ecology, Zoology, Plant sciences

## Abstract

Vector-borne pathogens frequently modify traits of their primary hosts and vectors in ways that influence disease transmission. Such effects can themselves be altered by the presence of other microbial symbionts, yet we currently have limited understanding of these interactions. Here we show that effects of pea enation mosaic virus (PEMV) on interactions between host plants and aphid vectors are modulated by the presence of different aphid endosymbionts. In a series of laboratory assays, we found strong interactive effects of virus infection and endosymbionts on aphid metabolomic profiles, population growth, behavior, and virus transmission during aphid feeding. Furthermore, the strongest effects—and those predicted to favor virus transmission—were most apparent in aphid lines harboring particular endosymbionts. These findings show that virus effects on host-vector interactions can be strongly influenced by other microbial symbionts and suggest a potentially important role for such interactions in disease ecology and evolution.

## Introduction

Because vector-borne disease transmission depends critically on the ways in which vector organisms interact with infected and uninfected hosts [1–3], pathogens can be selected to influence these interactions by altering relevant vector and host traits [4]. Insect-borne plant viruses, for example, have been shown to alter host-plant quality for vectors, as well as plant traits that influence vector recruitment [3, 5–10]. Once acquired by a vector, these viruses can also directly manipulate sensory responses and host-seeking behaviors [11–13]. Such pathogen effects are likely to be influenced by the presence of other microbial symbionts of the host or vector, whose fitness interests may diverge from those of pathogens, and which can also modify relevant host and vector traits [14–17]. Indeed, endosymbionts associated with insect vectors are known to influence reproductive rates, behavioral patterns, and other traits with relevance for disease transmission [18–24]. However, relatively little work has examined how the presence of such symbionts modulates pathogen effects on host-vector interactions [17, 25]. Moreover, the interface between disease transmission and microbe ecological interactions remains an emerging field [17, 25].

Aphids form well-documented associations with obligate and facultative endosymbionts and are also important vectors of plant pathogens, including viruses that can have dramatic effects on aphid-plant interactions [2, 3, 17, 18, 25, 26]. Such effects can be mediated by virus-induced changes in plant traits such as defense chemistry, nutritional quality, leaf color, and volatile emissions [9, 27, 28], which, in turn, can influence aphid recruitment to and dispersal from infected plants, as well as the likelihood of virus uptake during aphid feeding [5, 8, 10, 11, 29]. These plant-mediated effects can also influence aphid population growth and thus the rate at which infected aphids disperse and infect new hosts [30–32]. Once acquired by the aphid vector, viruses can also influence transmission via direct effects on vector traits such as locomotor activity and preferences for olfactory and visual cues associated with infected and uninfected host plants [12, 13].

Such direct manipulation of vector phenotypes can be more readily achieved by viruses that actively infect the vector (i.e., those that circulate within vector tissues rather than merely being physically transported on cuticular surfaces) both because they are well situated to influence vector physiology and because they experience more intense selection for adaptation to the environment provided by the vector [33], plausibly including the presence of other microbial symbionts [17, 25]. Plant viruses that form such intimate associations with their arthropod vectors are said to be persistently transmitted, in contrast to non-persistently transmitted viruses that form only transitory associations with vectors (e.g., via mechanical attachment to vector mouth parts) [2, 26]; a further distinction can be made between persistent viruses that merely reside within the salivary glands or other vector tissues (nonpropagative viruses) and those that actively replicate within the vector (propagative) [2, 26]. Persistent viruses are frequently transmitted by only a small number of closely related vector species and exhibit high levels of adaptation to transmission by specific vectors [1, 33].

In aphids, the reliable presence of specific endosymbionts is likely an important feature of the vector environment that shapes the evolution of persistently transmitted viruses [17, 25]. Obligate nutritional symbiosis with endobacteria (typically *Buchnera aphidicola*) is a key feature of aphid biology that provides access to essential amino acids absent in the phloem diet [18, 34]. Most aphids also harbor facultative endosymbionts, which can have diverse effects on aphid traits and interactions with other organisms, including resistance to pathogens and parasitoids [reviewed in [35]]. Facultative symbionts have also been shown to influence aphid interactions with host plants [18, 36, 37], including via effects on traits such as feeding behavior [19] and population growth [38, 39], which, in turn, can influence virus transmission [5, 32]. To date, however, limited research has investigated interactions between endosymbionts and plant viruses, with most studies focusing on direct effects of symbionts on virus circulation within the vector [17, 25].

A few previous studies have reported positive effects of facultative endosymbionts on the efficiency of virus transmission by whiteflies [40–42], while another reported negative effects on the transmission of a plant virus by planthoppers [43]. However, only two previous studies have investigated effects of endosymbionts and viruses on vector traits, both focusing on aphid vectors and non-persistent viruses [23, 44]. The first reported that aphid feeding on plants infected by cucumber mosaic virus led to the decline in the population of obligate endosymbiont within the aphid, which the authors speculated might influence rates of aphid dispersal to healthy plants via nutritional effects [44]. In the second study, the presence of different facultative endosymbionts (*Hamiltonella defensa* or *Arsenophonus* sp.) altered the frequency with which aphid vectors probe tissues of plants infected by watermelon mosaic virus, with potential implications for virus acquisition and dispersal by aphid vectors [23]. These studies provide initial evidence that endosymbionts may modulate the manipulative effects of plant viruses on host-vector interactions. As discussed above, there is also reason to suspect that interactions with endosymbionts might be more pronounced in the case of persistently transmitted viruses. However, little or no previous work has explored how the presence of aphid endosymbionts influences the effects of persistently transmitted viruses on vector interactions with host plants. In particular, we are not aware of previous work exploring the relevance of endosymbiont-virus interactions for critical features for virus transmission and epidemiology, including vector reproductive traits, host-seeking behaviors, and rates of dispersal from infected to uninfected plants.

The current study explores how the presence of different facultative endosymbionts influences the transmission of a persistently transmitted non-propagative (+ssRNA) plant virus, pea enation mosaic virus 1 (PEMV), as well as the separate and combined effects of virus infection and endosymbionts on aphid performance, metabolomics, and transmission-relevant behavioral traits. In a series of laboratory experiments, we investigated effects of PEMV on interactions between genetically uniform pea aphid lines (*Acyrthosiphon pisum* clone LSR1) harboring different endosymbiont sets and fava bean plants (a “universal” host that can be colonized by pea aphids of any background). Our results show that virus effects on plant-vector interactions can be differently influenced by endosymbiont species and strains, and thus highlight the potential importance of such microbial interactions for vector-borne disease transmission.

## Results

### Effects of PEMV infection on plant defense and nutrition

**Fig. 1:**
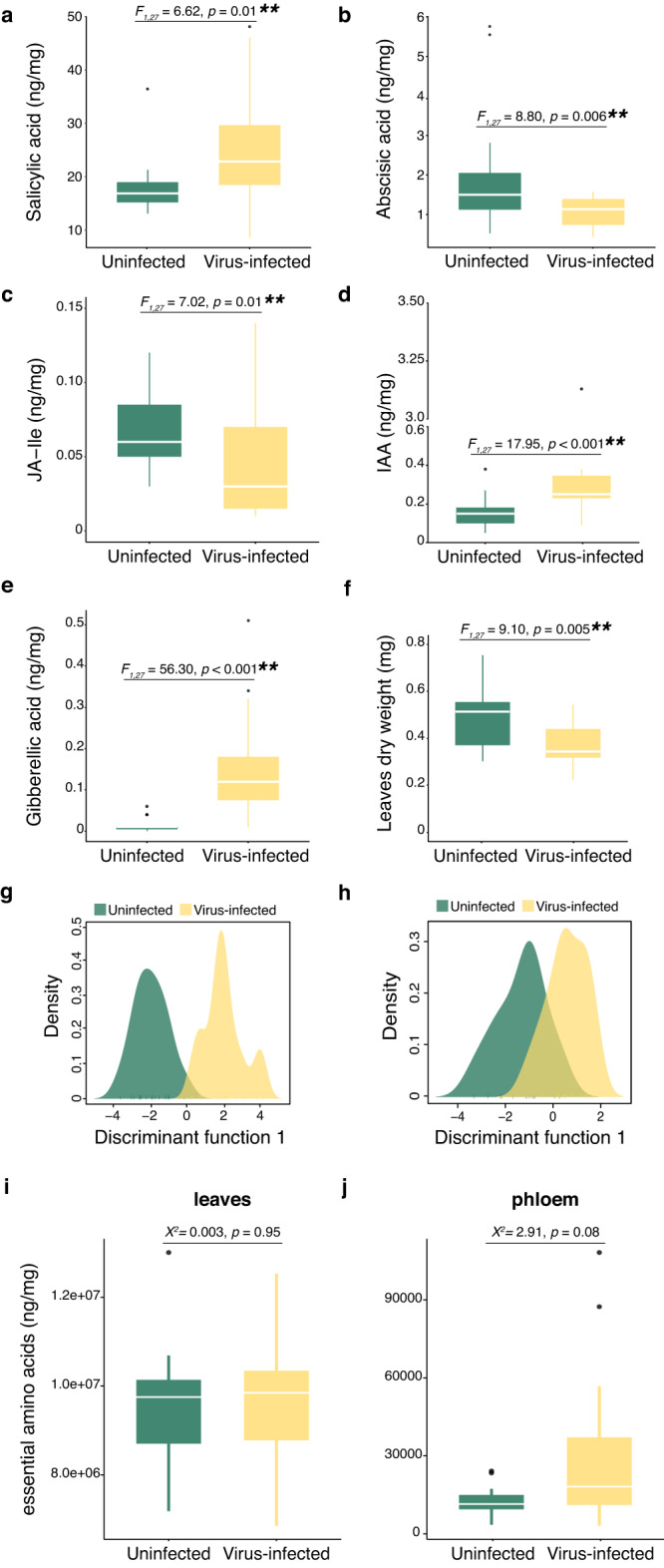
Phenotypic traits of uninfected and virus-infected plants. **a**–**e** Phytohormones levels and (**f**) leaves biomass of uninfected and plants infected with pea enation mosaic virus (PEMV); nonparametric ANOVA, aligned rank transform (**p ≤ 0.01; *N* = 14). JA-Ile: jasmonic acid-isoleucine; IAA: acid indole acetic. **g** Density plot using the first discriminant function to differentiate metabolomic profile of leaves from uninfected and PEMV-infected plants; PERMANOVA (*pseudo-F* = 1.86*, R*^2^  =  *0.062, p* = 0.15, *N* = 14). **h** Density plot using the first discriminant function to differentiate metabolomic profile of the phloem from uninfected and PEMV-infected plants; PERMANOVA (*pseudo-F* = 0.67*, R*^2^  = *0.026, p* = 0.59; *N* = 14). **i** Levels of essential amino acids in the leaves and (**j**) in the phloem of uninfected and PEMV-infected plants; nonparametric ANOVA, generalized linear mixed models (*N* = 14). Boxplots display median line, interquartile range (IQR) boxes, 1.5 × IQR whiskers; points indicate outlier observations.

Our initial experiments explored how PEMV infection influences plant traits that might be expected to mediate interactions with aphid vectors. Chemical analysis of plant hormones revealed that infected plants had elevated levels of salicylic acid, a key signaling molecule in pathways involved in plant defense against pathogens (Fig. 1a), but reduced levels of abscisic acid, which mediates pathways involved in defense against herbivory and abiotic stress (Fig. 1b). While PEMV infection had no effect on the levels of several other hormones implicated in general plant resistance against herbivory (Linolenic acid, 12-oxophytodienoic acid, and jasmonic acid; Fig. S1), infected plants had reduced levels of the isoleucine conjugate of jasmonic acid (Fig. 1c), which can mediate plants resistance specifically against aphids [45]. Infection did not influence the growth-promoting phytohormone trans-Cinnamic acid (Fig. S1) but increased levels of acid indole acetic (IAA, Fig. 1d) and gibberellic acid (Fig. 1e), which play roles in growth coordination and the development of plant organs. Consistent with these findings, PEMV-infected fava beans exhibited reduced biomass (Fig. 1f). Finally, levels of most nutritional metabolites in PEMV-infected plants were broadly similar to those of uninfected plants (Fig. 1g, h and S1; Table S1). Overall levels of essential amino acids in the phloem of PEMV-infected plants were higher than uninfected plants, but this result was statistically insignificant for comparisons in both leaf and phloem tissues (Fig. 1i, j).

### Effects of PEMV infection and endosymbionts on aphid performance

**Fig. 2:**
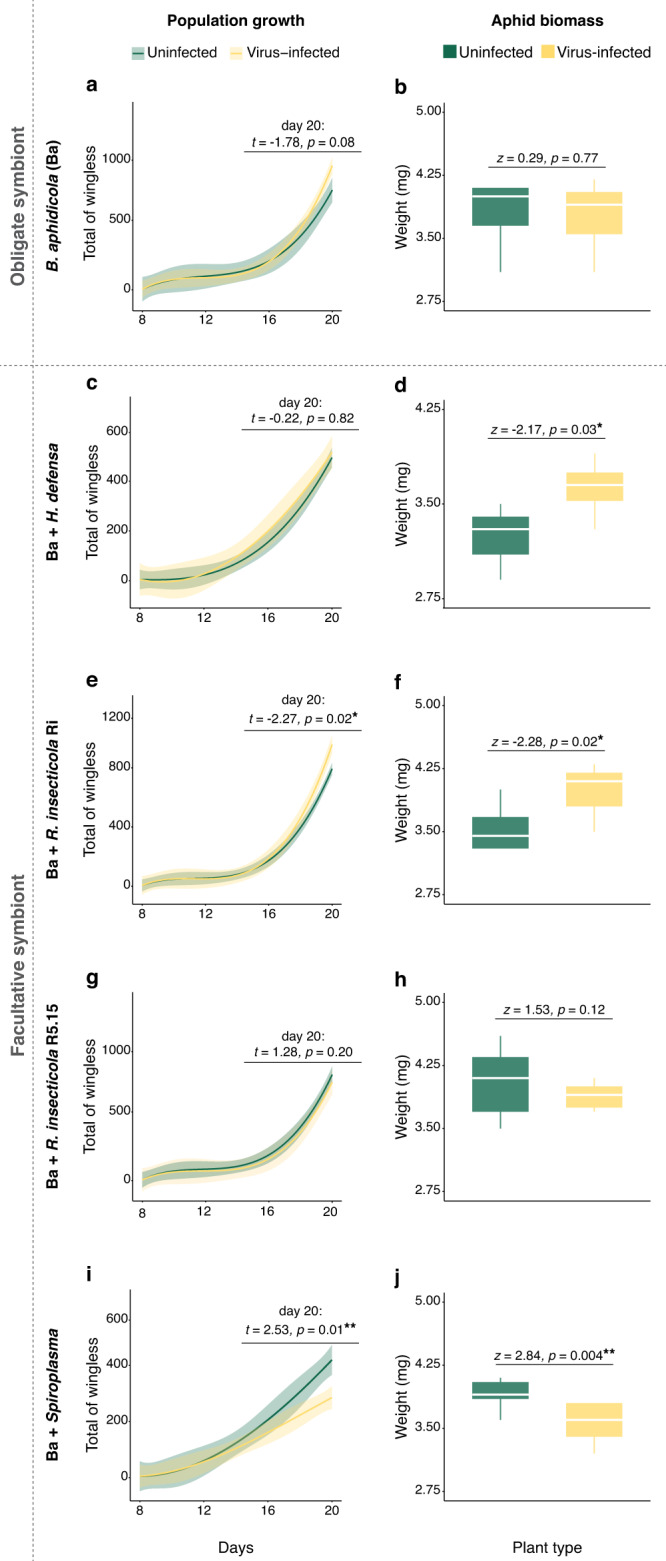
Performance of aphids harboring different symbionts and growing on uninfected or virus-infected plants. **a**, **c**, **e**, **g**, **i** Total of wingless population over time, and (**b**, **d**, **f**, **h**, **j**) biomass of wingless aphids on uninfected (green lines and boxplots) and plants infected with pea enation mosaic virus (yellow lines and boxplots). Generalized linear mixed models (**a**, **c**, **e**, **g**, **i** lines represent averaged population projection and shadowed area display 95% confidence interval; **b**, **d**, **f**, **h**, **j** boxplots display median line, interquartile range (IQR) boxes, 1.5 × IQR whiskers; **p ≤ 0.01 and *p < 0.05). Aphids differed in symbiont composition: only *B. aphidicola* (Ba) (a-b: uninfected *N* = 7, infected *N* = 8), Ba + *H. defensa* (**c**, **d**: uninfected *N* = 9, infected *N* = 9), Ba + *R. insecticola* strain Ri (**e**, **f**: uninfected *N* = 9, infected *N* = 9), Ba + *R. insecticola* strain R5.15 (**g**, **h**: uninfected *N* = 10, infected *N* = 8), and Ba + *Spiroplasma* (**i**, **j**: uninfected *N* = 7, infected *N* = 8).

To evaluate the combined effects of PEMV and endosymbionts on aphid performance, we tracked aphid population growth and biomass over three generations on uninfected and virus-infected fava beans. These experiments employed five clonal lines of pea aphids differing only in the specific endosymbionts present, including one line harboring only the obligate endosymbiont *Buchnera aphidicola* and four others harboring *B. aphidicola* in combination with one of four different facultative endosymbionts: *Hamiltonella defensa*, *Regiella insecticola* strain Ri, *R. insecticola* strain R5.15, or *Spiroplasma* sp. There was a strong interaction effect between virus infection status and endosymbionts on aphid population growth and biomass (Fig. 2) (GLMMs, interaction endosymbiont *vs*. virus infection, population growth: *X*^2^ = 9.68, *p* = 0.04; aphid biomass: *X*^2^ = 19.89, *p* < 0.001). Furthermore, all aphid performance traits we assessed were strongly influenced by endosymbionts but not by the virus alone (endosymbiont, *X*^2^ > 16.91, *p* ≤ 0.002; virus, *p* > 0.05), including the total number and weight of winged morphs (Figs. S2, S3). Aphids harboring only the obligate endosymbiont *B. aphidicola* performed similarly on infected and uninfected plants (*B. aphidicola:* Fig. 2a, b), as did those also harboring the facultative endosymbiont *R. insecticola* strain R5.15 (Fig. 2g, h). However, all other endosymbiont lines exhibited significant variation in performance on plants differing in infection status. Aphids harboring *Spiroplasma* performed worse on PEMV-infected plants than on uninfected plants (Fig. 2i, j), while aphids harboring either *H. defensa* (Fig. 2c, d) or R*. insecticola* strain Ri (Fig. 2e, f) performed better. The latter two lines also exhibited the worst performance (lowest biomass gain) among all aphid lines on uninfected plants (Figs. 2 and S4), although the difference in biomass gained by aphids harboring *R. insecticola* strain Ri did not differ statistically from those harboring only *B. aphidicola* (Fig. S4).

### Effects of PEMV infection and endosymbionts on aphid behavior

To explore the effects of virus infection and endosymbionts on aphid behavior and host-plant preferences, we employed a dispersal assay (performed in combination with the performance assay described above), as well as a separate dual-choice experiment examining aphid preferences for infected and uninfected plants. These two assays have the potential to capture different features of aphid behavior, as the first assesses the dispersal rate and plant preferences of aphids that chose to leave an initial host, while the latter assesses preferences of aphids that were forced to make a choice.

**Fig. 3:**
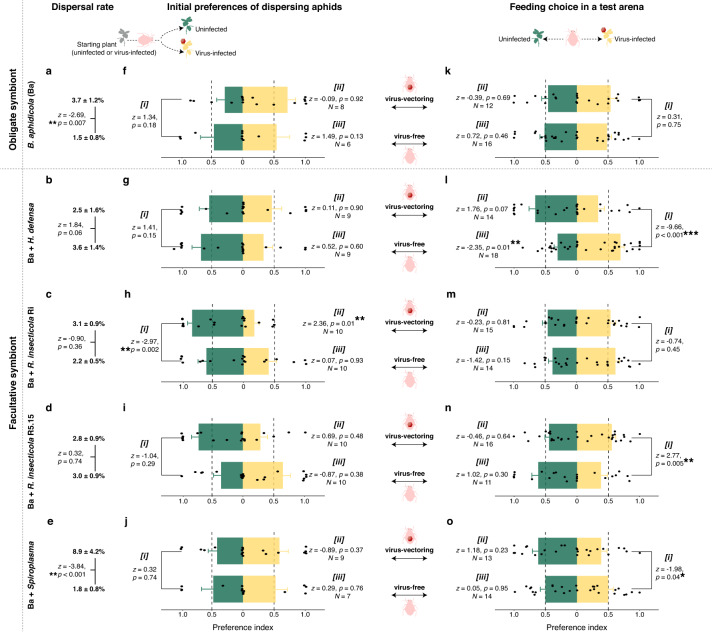
Plant preferences of virus-free and virus-vectoring aphids harboring different sets of endosymbionts. **a**–**e** Dispersal rate (average ± standard error) of aphids according to their virus status. Generalized linear mixed models (GLMMs; connecting line represents statistical comparison between dispersal rate of virus-free and virus-vectoring aphids with the same symbiont; **p ≤ 0.01). **f**–**j** Preferences between uninfected (green bars) and PEMV-infected plants (yellow bars) of dispersing aphids and (**k**–**o**) of aphids in a test arena. GLMMs (bars indicate the mean choice, error bars denote standard errors, and dots indicate replicates; ***p < 0.0001, **p ≤ 0.01, *p < 0.05). In (**f**–**o**), [***i***] represents statistical test for effects of virus status (virus-free vs virus-vectoring) on host plant preferences (uninfected *vs* PEMV-infected) of aphids with the same symbiont; [***ii***] corresponds to test of host plant preferences (uninfected vs PEMV-infected) by virus-vectoring aphids with the same symbiont (upper bars and values in each panel); and [***iii***] indicates test of host plant preferences (uninfected *vs* PEMV-infected) by virus-free aphids with the same symbiont (lower bars and values in each panel). Aphids differed in symbiont composition: only *B. aphidicola* (Ba) (**a**, **f**, **k**), Ba + *H. defensa* (**b**, **g**, **l**), Ba + *R. insecticola* strain Ri (**c**, **h**, **m**), Ba + *R. insecticola* strain R5.15 (**d**, **i**, **n**), and Ba + *Spiroplasma* (**e**, **j**, **o**).

In the first assay, each replicate aphid colony was given access to two nearby plants differing in infection status (see Methods, Fig. S5). Target plants were checked daily, and we recorded aphid plant preferences and initial dispersal rate (total of dispersed aphids by the total population on the day dispersal was first observed). The timing of initial dispersal was not significantly influenced by infection status, endosymbionts, or their interactions (Fig. S6). However, both the dispersal rate and host-plant preferences of dispersing aphids showed strong interactive effects of infection status and endosymbionts (Fig. 3a–j) (GLMM, interaction endosymbiont *vs*. virus, dispersal rate: *X*^2^ = 22.27, *p* < 0.001; plant choice: *X*^2^ = 13.29, *p* = 0.009). The presence of the virus was associated with higher rates of dispersal for aphids harboring only the obligate symbiont *B. aphidicola*, (Fig. 3a), as well as for those also harboring the facultative endosymbiont *Spiroplasma* (Fig. 3e), but not for aphid lines harboring other facultative symbionts (Fig. 3b–d). Infection status had no significant effects on host-plant preferences for aphids that harbored only the obligate symbiont or those harboring most of the facultative endosymbionts, including *Spiroplasma* (Fig. 3f, g and i, j). For aphids harboring *R. insecticola* strain Ri, however, the presence of the virus was associated with a strong dispersal preference for uninfected plants (Fig. 3h*i–ii*).

In the second behavioral assay, we presented virus-free and virus-vectoring aphids from each endosymbiont line with host plants differing in infection status in a dual-choice arena, and assessed their plant preferences (see Methods, Fig. S5). The results of this assay also indicate that the interaction of virus infection status and endosymbionts has strong effects on aphid preferences (Fig. 3k–o) (GLMM, interaction endosymbiont *vs*. virus, *X*^2^ = 78.90, *p* < 0.001). As in the previous dispersal assay, aphids harboring only the obligate endosymbiont *B. aphidicola* exhibited no preference between virus-infected and uninfected host plants regardless of their own virus status (Fig. 3k). In contrast to the dispersal assay, infection status also did not influence host preferences of aphids harboring *R. insecticola* strain Ri (Fig. 3m), but did affect the host-plant preferences for aphids harboring three other facultative endosymbionts: virus-free aphids harboring *H. defensa* exhibited a preference for infected plants (Fig. 3l*iii*) that shifted toward healthy plants for virus-vectoring aphids (Fig. 3l*i*); virus infection produced a similar preference shift in aphids harboring *Spiroplasma* (Fig. 3o*i*), but a shift in the opposite direction for those harboring *R. insecticola* strain R5.15 (Fig. 3n*i*).

### Effects of PEMV infection and endosymbionts on aphid metabolomic profiles

**Fig. 4:**
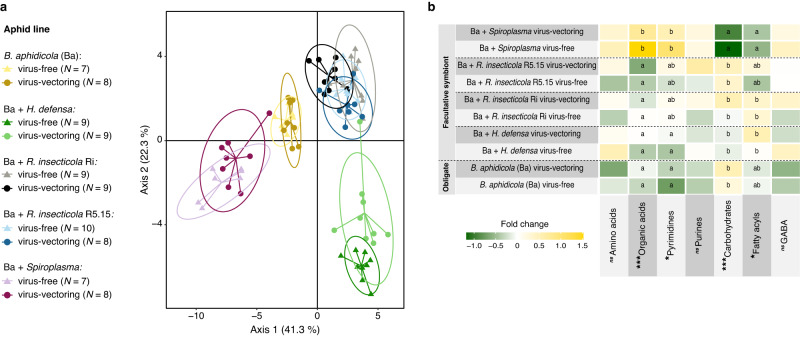
Metabolomic profile of virus-free and virus-vectoring aphids harboring different sets of endosymbionts. **a** Discriminant analysis of principal components showing separation of aphid lines based on endosymbiont and virus vectoring status. **b** Heatmap representing the fold changes of metabolite groups in aphid lines; GABA: gamma-aminobutyric acid. The effects of endosymbionts and virus vectoring status on the levels of metabolite groups were tested using nonparametric ANOVAs (***p < 0.001, *p < 0.05, and **ns** indicates *p* > 0.05; same letters do not differ according to subsequent Tukey’s pairwise comparisons): see Supplementary Table 2 for detailed statistical results.

We analyzed the metabolomic profile of aphids in our performance assay to gain insight into potential mechanisms underlying the observed effects of endosymbionts and virus-vectoring status on aphid biology and behavior. These analyses also revealed strong interaction effects of endosymbionts and virus infection (Fig. 4 and S7) (PERMANOVA, interaction endosymbiont vs. virus, *pseudo-F* = *2.25, R*^2^ = *0.21, p* = 0.003).

While virus infection alone did not contribute to treatment grouping (PERMANOVA, *pseudo-F* = 0.33, *R*^2^ = 0.004*, p* = 0.89), aphid metabolomic profiles showed marked clustering based on their endosymbiotic bacteria (PERMANOVA, *pseudo-F* = 4.29, *R*^2^ = 0.17*, p* = 0.001), which are apparent in comparisons of biologically relevant group of metabolites across different aphid lines (Fig. 4b, Table S2). Aphids harboring the obligate symbiont *B. aphidicola* and most facultative symbionts exhibited similar levels of the majority of tested compound classes; however, the aphid line harboring *Spiroplasma* exhibited the highest levels of molecules involved in carbon and nucleotide metabolism, such as organic acids and pyrimidines, and the lowest levels of compounds relevant for energy and lipid metabolism, such as carbohydrates and fatty acyls (Fig. 4b, Table S2).

Comparison of compound levels within each line revealed varied effects of virus infection (Fig. S7). While virus infection status did not exhibit strong effects on the metabolic profiles of most aphids, differences in the levels of several amino acids between virus-vectoring and virus-free aphids harboring *H. defensa* were apparent, and a similar pattern was observed for carbohydrates in aphid lines harboring *R. insecticola* strain Ri (Fig. S7). For lines harboring *R. insecticola* strain Ri, our metabolomic analyses also revealed differences in levels of gamma-aminobutyric acid (GABA), a compound involved in olfactory associative memory, with virus-vectoring aphids exhibiting significantly higher levels of this compound than virus-free individuals (Fig. 4b and S7) (*X*^2^ = 4.23, *p* = 0.03).

### Endosymbiont effects on virus transmission

**Fig. 5:**
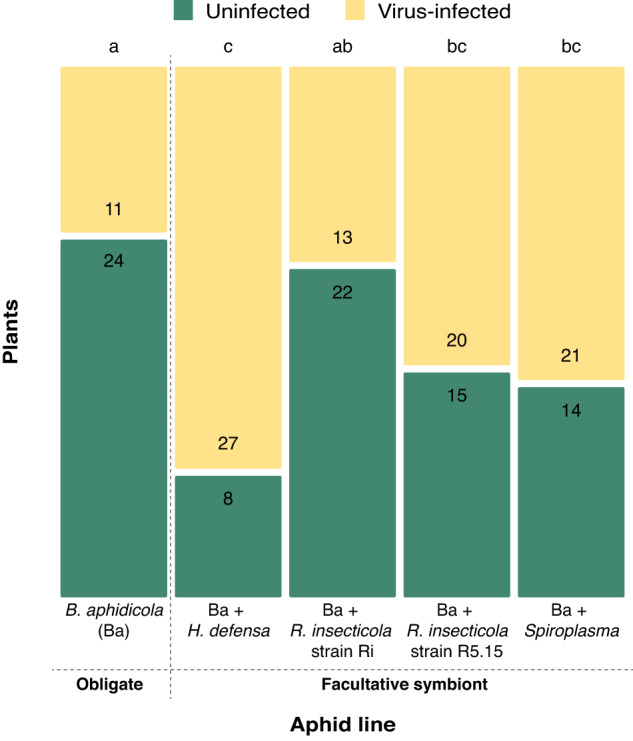
PEMV transmission by aphids harboring different sets of endosymbionts. Numbers inside the green bars indicate total of plants that were not infected while numbers inside the yellow bars represent total of plants that became infected with pea enation mosaic virus (PEMV) according to the composition of endosymbionts in the aphid vector (bottom labels). Generalized linear mixed model (*N* = 35). Same letters (top of the bars) do not differ according to multiple group comparisons with Tukey’s test.

We examined the effects of endosymbionts on virus transmission by placing individual virus-vectoring aphids from our experimental lines on uninfected plants for a controlled period and then assessing rates of plant infection. This design is inherently conservative, as it excludes possible effects mediated by aphid behavioral preferences (e.g., enhanced attraction of virus-vectoring aphids to healthy plants) or performance (e.g., increased population growth on and dispersal from infected plants). Nevertheless, our results reveal strong endosymbiont effects on rates of virus transmission (Fig. 5) (GLMM, endosymbiont: *X*^2^ = 21.98, *p* < 0.001). We found significantly higher rate of virus transmission for aphids harboring *H. defensa* compared to those harboring only the obligate endosymbiont *B. aphidicola* or those also harboring the facultative symbiont *R. insecticola* strain Ri, while aphids harboring *R. insecticola* R5.15 or *Spiroplasma* exhibited intermediate levels of transmission (Fig. 5).

## Discussion

In addition to documenting diverse effects of endosymbionts and virus infection on aphids, our results reveal strong interactions between infection status and the presence of specific endosymbiont strains (Summary Table S3). In particular, the facultative endosymbionts *R. insecticola* strain Ri and *H. defensa* exhibited the strongest interactions with PEMV, generally influencing aphid phenotypes in ways that would be predicted to enhance virus transmission. For example, aphids harboring these strains exhibited significantly enhanced performance on PEMV-infected plants, despite performing worse than other aphid lines on uninfected plants (Fig. 2d–f, Fig. S4). Virus infection status also affected the host-plant preferences of aphids harboring these endosymbionts, with initial preferences of virus-free aphids for PEMV-infected plants being reversed for aphids vectoring the virus in either the dispersal (*R. insecticola* strain Ri, Fig. 3h) or feeding-choice assay (*H. defensa*, Fig. 3l). Furthermore, our metabolomic analyses revealed interactions with virus-infection status that might be related to the altered behavior and performance observed for these lines, including changes levels of key sugars, amino acids, and, in the case of *R. insecticola* strain Ri, a compound (GABA) previously shown to influence olfactory memory (Fig. 4, Fig. S7) [46]. Finally, results from our transmission assays revealed significantly higher rates of PEMV transmission by aphids harboring *H. defensa* (but not *R. insecticola* strain Ri) relative to the line harboring only the obligate endosymbiont (Fig. 5). Taken together, these results reveal variation in virus effects on aphids harboring different facultative endosymbionts, and on their interactions with host plants.

Vector acquisition of persistently transmitted plant viruses such as PEMV typically requires sustained aphid feeding on infected plant tissues, and numerous studies have reported virus effects on host plant traits that tend to prolong feeding [2, 3, 26]. In our experiments exploring virus effects on plant traits, PEMV infection reduced defenses against herbivores and biomass (Fig. 1a–f). In addition, levels of essential amino acids—a limiting dietary resource for aphids [34, 47]—in the phloem of PEMV-infected plants were higher than levels in uninfected plants, although this trend was statistically insignificant (Fig. 1j). Negative effects on growth are typical for plant pathogenic viruses [28], while effects on plant defenses and amino-acid availability are consistent with previously reported effects of persistently transmitted viruses on host plant chemistry that appear favorable for transmission [2, 3, 26].

The presence of endosymbionts can influence host-plant chemistry [20, 36, 37], as can aphid herbivory itself [48, 49], and such effects can influence aphid performance and other traits relevant to virus transmission [21, 29, 30, 50]. While some previous studies reported no effects of PEMV infection of host plants on aphid performance traits such as fecundity [29, 30], results from our performance assay indicate that such effects can be conditional on the presence of particular endosymbionts (Fig. 2). Indeed, we observed a range of virus effects on population growth and biomass for aphid lines harboring different endosymbionts (Fig. 2), including no effects on performance (only *B. aphidicola* (Ba): Fig. 2a, b; Ba + *R. insecticola* strain R5.15: Fig. 2g, h); reduced performance (Ba + *Spiroplasma*: Fig. 2i, j); and enhanced performance (Ba + *H. defensa*: Fig. 2d; or Ba + *R. insecticola* strain Ri: Fig. 2e, f). Aphid lines harboring *H. defensa* and *R. insecticola* strain Ri exhibited relatively low levels of biomass accumulation on uninfected plants (Fig. S4), and previous studies have reported adverse effects of these endosymbionts on aphid performance [51, 52]. The current findings thus suggest that such negative effects may be mitigated or reversed in environments where virus-infection of host plants is prevalent.

Numerous studies have reported apparently manipulative effects of plant virus on vector behaviors and plant-vector interactions, including enhanced attraction of virus-free vectors to infected host plants, followed by a reversal of preferences for healthy vs infected plants once the vector acquires the virus [8, 10, 12, 13]. The results of our behavioral assays provide the first evidence that such behavioral effects can be conditional on the presence of particular endosymbionts within the vector (Fig. 3). Furthermore, the combined effects of virus and endosymbionts showed some consistent patterns across our performance and plant preferences assays (Fig. 2, Fig. 3). For example, for aphids harboring only the obligate endosymbiont *B. aphidicola*, virus infection had no significant effects on either performance or feeding preferences (Figs. 2a, b, 3f,k), while all aphid lines harboring facultative endosymbionts exhibited significant effects of virus-infection on host-plant preference in at least one of our behavioral assays (Fig. 3). As noted above, aphid lines harboring *H. defensa* and *R. insecticola* Ri performed better on infected than on uninfected plants (Fig. 2d–f, Fig. S4); yet, the significant behavioral effects of infection observed for these lines were characterized by a shift toward preference for uninfected plants by aphids vectoring the virus (*H. defensa*: Fig. 3l*i*; *R. insecticola* Ri: Fig. 3h*i*), which would appear favorable for virus transmission despite being inconsistent with the observed positive effects of infection on aphid performance. Meanwhile, lines harboring *Spiroplasma* exhibited a similar, if less pronounced, behavioral shift in our second behavioral assay (Fig. 3o*i*), while virus infection had negative effects on aphid performance for this line (Fig. 2i, j) as well as strong positive effects on dispersal (Fig. 3e). Finally, *R. insecticola* R5.15 exhibited the opposite behavioral shift in our preference assay (Fig. 3n*i*), while the performance of this aphid line was not affected by the infection status of host plants (Fig. 2g, h).

Our aphid metabolic assays also revealed a broad range of separate and interactive effects of endosymbionts on biologically relevant compound groups, such as organic acids, pyrimidines, carbohydrates, and fatty acyls (Fig. 4 and S7). In insects, these compounds mediate processes that influence growth, reproduction, and resilience against plant defense, including energy availability, chitin synthesis, and detoxification [53–56]. Furthermore, virus effects on the improved performance of some aphid lines might be explained by increased levels of limited resources for aphids, especially amino acids [47]. For example, aphids harboring *H. defensa* had increased biomass in the presence of the virus (Fig. 2d), as well as increased levels of some amino acids (Fig. S7). Correspondingly, aphids harboring *R. insecticola* strain Ri exhibited improved population growth and biomass in the presence of the virus (Fig. 2e, f), as well as increase levels of some carbohydrates such as trehalose (Fig. S7) that can act as source of instant energy in insects [57]. In addition, virus-vectoring aphids from lines harboring *R. insecticola* strain Ri, which as discussed above exhibited behavioral patterns that appear conducive to virus transmission but inconsistent with virus-effects on host-plant quality, exhibited increased levels of gamma-aminobutyric acid (GABA; Fig. 4b and S7), a modulatory compound shown to negatively impact olfactory-associated preferences in *Drosophila* [46]. Previous findings indicate that the GABAergic system can be independently modulated by both endobacteria and vectored viruses through diverse mechanisms that are not fully understood, including via indirect effects on the levels of precursors in the insect diet (e.g., glutamic acid) or direct stimulation and synthesis of glutamate and linked pathways in the insect gut [58, 59]. Given that only aphids harboring *R. insecticola* strain Ri and vectoring the virus had elevated levels of GABA, it is unlikely that this endosymbiont or PEMV individually influenced GABA via these former mechanisms, and their combined proximate effects on GABA induction in pea aphids remains to be investigated.

The possibility that PEMV might alter aphid behavior in the presence of particular endosymbionts (e.g., via effects on GABA) is plausible in light of previous reports that other vector-borne viruses can induce similar behavioral changes favoring transmission, potentially by exploiting cellular machinery of insect vectors [11, 13], and specifically those mediating immune responses [33]. For example, tomato yellow leaf curl virus triggers apoptosis in the brain of whitefly vectors that impair visual and olfactory systems [11, 13], resulting in a reduced preference for infected vs. healthy plants [13]. As our results indicate that virus direct effects on vector behaviors are modulated by the presence of endosymbionts, virus effects on vector traits might also plausibly be facilitated by endosymbionts already exploiting innate immune responses of insect vectors. Accordingly, recent studies have shown that aphid symbiosis with both *H. defensa* and *R. insecticola* (but not other endosymbionts) is maintained by alteration of insect innate immunity against microbes, including via effects on defensive peptides and enzymes involved in processes such as autophagy and apoptosis (e.g., antimicrobial peptides-AMPs and lysozymes) [60, 61] that can modulate virus-induced effects on insect vector behavior [33]. While our results indicate combined effects of specific symbionts and the virus on metabolomic environment within the vector, further investigation of the transcriptome and immune responses of pea aphids may yield new insight into the mechanisms by which these microbes modulate vector behavioral traits favoring transmission.

Our transmission assay demonstrates that the presence of particular facultative endosymbionts can indeed influence rates of virus transmission to previously uninfected host plants during aphid feeding. Previous work suggests that endosymbionts may affect virus circulation within aphid vector or the attenuation of plant defenses by aphid feeding (e.g., via salivary proteins), which, in turn, might influence infection rates of novel host plants [17, 25, 36, 37]. In the current study, the highest rates of transmission were observed for aphid lines harboring *H. defensa* (Fig. 5), for which we also observed significant effects of infection on aphid performance (Fig. 2d) and behavior (Fig. 3l). In contrast, the lowest rates of transmission were observed for aphids harboring only the obligate endosymbiont *B. aphidicola*, while lines harboring other facultative endosymbionts generally exhibited intermediate levels of transmission (Fig. 5). A recent study documented that *H. defensa* (but not other symbionts) facilitates susceptibility to a viral pathogen of aphids (a +ssRNA virus like PEMV), including via effects on virus titers [62]. Considering the latter study and our findings, it is possible that *H. defensa* influences viral recognition or permissibility within the aphid, which can modulate levels of virus acquisition or retention in relevant tissues for subsequent transmission (e.g., salivary glands). It bears noting, however, that the simplified design of our transmission assay is inherently conservative, obscuring any potential role of the behavioral and performance effects observed in previous assays in mediating differences in transmission. Although challenging to accomplish [26, 63], studies examining effects on transmission under more natural conditions should be a priority for future research exploring the separate and combined effects of pathogenic viruses and endosymbionts on host-vector interactions in this and other pathosystems.

In conclusion, our findings point to a potentially important role for aphid endosymbionts in modulating virus effects on host-vector interactions. We report diverse effects of endosymbionts and PEMV on a range of behavioral and other aphid traits, as well as interactive effects of virus infection and endosymbionts that in some cases appear conducive to transmission (especially for aphids harboring *H. defensa* and *R. insecticola* strain Ri); moreover, we observed increased rates of transmission during feeding by PEMV-infected aphids harboring *H. defensa*. These findings add to a growing literature demonstrating that the microbiome is a key player in host physiology, performance, and behavior [18, 19, 34, 38, 39]. As aphid populations are generally heterogenous for facultative symbionts [64], these results further suggest that endosymbiont diversity is a potentially important factor influencing host-vector interactions that should be considered when investigating transmission-relevant traits of aphid-borne viruses. More generally, these findings highlight the likely importance of interactions among pathogens and other microbial symbionts in the ecology and evolution of vector-borne diseases.

## Methods

### Study system

Pea aphids (*A. pisum*) LSR1 clones were kindly provided by Christoph Vorburger from the Evolutionary Ecology group, EAWAG, Switzerland. Four out the five lines were already established at EAWAG: lines harboring only *Buchnera aphidicola*, or this symbiont combined with *Regiella insecticola* strain Ri (also known as strain LSR1), *R. insecticola* strain R5.15, and *Spiroplasma* strain S161. The aphid line harboring *Hamiltonella defensa* strain 5A was created by microinjecting hemolymph from a donor aphid (clone 5A-T) into a receiver LRS1 clone carrying only *Buchnera aphidicola* according to methods described previously [65]. Aphid colonies were maintained in the laboratory on potted uninfected or PEMV-infected beans enclosed by permeable cellophane bags (185 mm width × 390 mm length; Celloclair AG, Switzerland) and under controlled abiotic conditions (22 °C, 60% relative humidity, 16:8 h light:dark photoperiod). Aphids were employed in the assays at least ten generations after microinjections. Presence of endosymbionts were confirmed before every experiment by caring out high salt extractions of aphid’s DNA [66], followed by PCRs diagnostic as described previously [67] (Table S4 for primers details).

PEMV was included in this study given that it is a well-documented virus system primarily vectored by pea aphids. This virus is considered to be a complex mainly consisting of two single-stranded positive sense RNAs (PEMV-1: genus *Enamovirus* of family Solemoviridae; PEMV-2: genus *Umbravirus* of family Tombusviridae) that predominantly infects leguminous host plants (Fabaceae) [68, 69]. Symptoms of host plants infected with PEMV include mosaic, stunting, hyperplastic outgrows, malformed pods, which ultimately leads to reduced yields of agriculture crops and economical losses [70, 71]. Some PEMV isolates can be mechanically transmitted while others are only transmitted by aphids in a persistent circulative and non-propagative manner [71]. Apart from the main vector pea aphids, several other aphid species have been reported to transmit this virus, including *Macrosiphum euphorbiae*, *Myzus persicae*, *M. ornatus*, *A. solani*, *A. gossypii*, and *Aulacorthum solani* [68]. During feeding, PEMV can be ingested by aphids with the phloem sap of infected host plants [71]. Within the aphid vector, virions are trans-cellularly transported through the gut into the hemocoel, and subsequently transported through the hemolymph into the salivary glands until, eventually virions are excreted with the saliva into a new host plant during aphid feeding [68, 69].

Pea aphids carrying PEMV were kindly provided by Sanford Eigenbrode from Chemical Ecology group at University of Idaho, USA. These aphids were placed on fava beans seedlings to generate the initial virus-infected source plants. Subsequently, plants were kept aphid-free until the first PEMV symptoms and confirmation of infection with an ELISA test (Nano Diagnostics, AR, USA). A subset of aphids from the line harboring only *B. aphidicola* was used to establish virus-free and virus vectoring colonies necessary for producing experimental plants. Although rearing aphid lines on PEMV-infected plants potentially introduces indirect (plant-mediated) virus effects on aphid biology and on virus acquisition by these insect vectors, plant viruses that are not vertically transmitted through their vectors (e.g., absent ovarial transmission) are only acquired upon vector feeding on virus-infected host plants. Therefore, virus vectoring aphids from which line were established by introducing nymphs of virus-free aphids onto a single PEMV-infected plant.

### Experimental plants

Individual fava bean seeds (*Vicia faba* var. *Fuego*) were planted in rectangular plastic pots (9 cm length × 9 cm width × 10 cm depth) containing potting substrate (Substraat 2; Klasmann-Delmann GmbH, Germany) mixed with 1 g of slow-release fertilizer (Osmocote Exact 5–6 M; NPK: 15-9-12). The pots were watered every second day and plants were grown in a climate chamber under controlled abiotic conditions (Kaelte3000, Switzerland; dimmable LED 4000 K lights at 170 μmol/s; 16:8 h photoperiod (light: dark) with 1 h of dawn and dusk; 22 °C as day and 20 °C as night temperature; 60% relative humidity). The seedlings were infected with PEMV after ten days of sowing (two leaf stage), by confining ten mixed instar virus vectoring aphids from *B. aphidicola* line on the plant with the air permeable cellophane bag. Uninfected plants were grown under the similar conditions, confined with virus-free aphids instead. Following a 4-day inoculation access period, aphids were removed from plants using a soft bristled paintbrush and the plants were maintained unbagged and aphid-free until they were used in experiments. Plants were employed after four weeks of virus inoculation in the preference assays and, after the experiment they were checked for PEMV using the ELISA test (Nano Diagnostics, AR, USA). Only positive virus-infected and negative uninfected plants pairs were kept in the final aphid preference data set. In all the other assays, plants were checked for PEMV before the experiments and after three weeks of virus inoculation. For that, one apical leaf of each uninfected and virus-infected plants was sampled and used in the ELISA test. Positive virus-infected and negative uninfected plants were employed in the bioassays one week after the test (four weeks after virus inoculation).

### Plant biomass and sampling

The top shoot of experimental plants was cut at the intersection with the first branch for an EDTA-facilitated phloem extraction carried through osmosis for 8 h (details in supplementary information). After this period, phloem samples were flash-frozen in liquid nitrogen and stored at −80 °C for further aqueous (polar) metabolites analysis.

We sampled the remaining plant tissue simultaneously to the incision for phloem collection. Plant leaves were collected in paper bags, flash-frozen in liquid nitrogen, and lyophilized for 48 h. The lyophilized leaves were weighted on the Mettler Toledo microbalance (±10 μg) and grounded to powder using three 3 mm round stainless-steel beads in 10 ml polypropylene tubes in a 2010 Geno/grinder (SPEX SamplePrep). From the homogenized tissue, two aliquots with 10–12 mg were weighted into a 2 ml safe-lock Eppendorf tube for extraction of phytohormones and aqueous metabolites.

### Phytohormone analysis

Plant hormones were recovered using a methanol extraction with isotope-labeled standards (d6-ABA, d5-JA, d4-SA, and d5-IAA; details in supplementary information). Following the extraction, 4 µl of each sample were injected into the Q-TOF LC-MS (Agilent Technologies 6550 iFunnel) with a RRHD Zorbax Eclipse Plus-C18 column (100 mm length, 2.1 mm diameter, 1.8 μm particle size). The solvent gradient used was 99% A (milli-Q water + 0.1% formic acid) to 99.5% B (acetonitrile + 0.1% formic acid) over 8 min with a flow rate of 0.6 ml/min. Final concentrations of free phytohormones were quantified in the MassHunter software relative to the recovery of their internal standard (SA, IAA, ABA, JA, and JA-Ile – the former quantified based on d5-JA) or relative to the calibration curve of unlabeled standards (CA, GA, α-LnA and OPDA). Amounts of phytohormones were also normalized by the dried weight of the plant sample.

### Aqueous metabolites of plants and aphids

The metabolites of leaves and aphid samples were extracted following an adapted version of the protocol described previously (details in supplementary information) [72]. Following the extractions, we injected 1 µl of each sample into a GC-MS (Agilent 7890B/5977 A GC-MSD, Agilent Technologies AG) equipped with a HP-5ms capillary column (30 m × 250 μm × 0.25 μm film thickness, Agilent Technologies AG). Helium was used as a carrier gas at a constant flow rate of 0.7 ml/min. The ionization method was Electron Impact (EI) in full scan mode. Mass spectra were measured at 70 eV and mass analysis ranged from 50 to 600 m/z. We set the inlet temperature to 250 °C and the split/splitless injector to pulsed splitless mode. The column temperature was kept at 70 °C for 5 min, raised at rate of 5 °C/min until it reached 325 °C, then kept at 325 °C for 2 min. Leaves and aphid samples were also injected using 10:1 split injector ratio for quantification of saturated compounds in the splitless run. Data for both MS and FID were collected simultaneously and analyzed using Mass Hunter Software (Agilent Technologies). Each compound was quantified based on the compound peak area relative to Ribitol. Compounds from samples were also normalized by dry weight of starting tissue. Compounds were identified by calculating the Kovats index, and by comparing their mass spectrum to the NIST 14 Mass Spectral Library and to available synthetic standards (Table S6 for compounds details).

### Aphid performance and first day of aphid dispersal

In a combined experiment, we evaluated behavioral patterns on the first day of aphid dispersal as well as aphid performance traits. For the former assessment, we monitored the timing until first day of aphid dispersal, choice of target host plants and rate of dispersing aphids (dispersing aphids / total of aphids in the population) (Fig. 3a–j) (Fig. S5). For the latter, we recorded aphid population growth over time and aphid weight on either uninfected or PEMV-infected plant treatment (Fig. 2).

For these experiments, two virus-free wingless adults from each aphid line were carefully placed on the leaf of an uninfected or virus-infected plant kept inside a fine-meshed cage (40 cm width × 60 cm length × 40 cm height) in the climate chamber. The aphids were allowed to lay offspring for 24 h and these adults were subsequently removed from the plants. After seven days (8th day from the start of the experiment), we kept only five of the founder nymphs to normalize the starting population throughout all replicates for the performance assay and, also at this point, we placed target host plant options for insect dispersal in the cage (Fig. S5).

To evaluate initial (first-day) aphid dispersal, one aphid-free uninfected and one PEMV-infected plant were positioned alongside and at the opposed cage extremity from the plant with the founder nymphs (40 cm apart) after 8 days from the beginning of this combined experiment assay. Cages were checked every day for tracking first dispersal event (timing of first dispersal), number of dispersed aphids on this first dispersal event, and target host plant choices (Fig. S5). This experimental design captures immediate aphid dispersal behavior as opposed to long-term dispersion patterns, providing information about potential effects of endosymbiont and virus on expediting insect willingness to disperse based on time course and population size. After the detection of the first dispersed aphids (in general, between 12 and 18 days after the start of the experiment; Fig. S6), we kept in the cage only the target host option belonging to the same treatment as the initial founder plant for continuity of aphid performance assay (e.g., only the target uninfected host plant option was kept in the cage with aphid population initially growing on uninfected plant, and vice-versa for PEMV-infected treatment: see Fig. S5). The dispersed aphids were also removed from the cages to avoid virus cross-contamination of host plants.

We assessed aphid performance by recording the total aphids every four days up to the 20th day after the birth of the founder aphids (until the beginning of third aphid generation; generation time 9 ± 2 days), as an attempt to best capture virus and endosymbionts effects on aphid traits. Aphids have trans-generational phenotypic plasticity, also referred as telescoping of generations because viviparous females bear developing embryos which themselves already contain embryos [73]; therefore, any grand-maternal and maternal physiological responses to the environment will conspicuously affect offspring phenotype. To account for effects of removing aphids dispersed to target plants distinct from the starting treatment (as mentioned above), we normalized aphid counts by the starting population before and after the dispersal, and this growth rate indices were used to compare population growths with and without correction for aphid removal (details below in “*Statistical analysis*”) (Table S5 indicates similar statistical output for normalized and non-normalized population count). In addition to population growth, aphid performance was also assessed by the weight of wingless and winged aphids on the 20th day. For that, aphids with the same morph type were weighted in groups of ten on a Mettler Toledo microbalance (±10 μg). In addition, groups of 20 aphids were sampled in a 2 ml Eppendorf tube, flash frozen in liquid nitrogen, lyophilized for 48 h, and stored in −80 °C freezer for extraction of aqueous metabolites (see methods above: “*Aqueous metabolites of plants and aphids”)*.

### Feeding choice of aphids in a test arena

In contrast to the dispersal assay, we evaluated the host plant preferences of aphids compelled to leave their initial host plant treatment (Fig. 3k–o). For that, we used a soft brush to induce aphids leaving their rearing plants via dropping behavior—a common behavioral strategy of pea aphids to flee from stressful conditions. Dropped wingless adults were grouped in tens and starved for 1 h prior the test. Thereafter, each group of ten aphids was inserted in the center of a petri dish arena (15 cm diameter). The top expanded leaf of one uninfected and one PEMV-infected plant were positioned on top of arena openings (2 cm diameter) so that olfactory, visual, and gustatory cues from each plant treatment were presented within the arena at opposite extremities (Fig. S5). A humidified cotton pad was placed on top of each leaf to secure its position and to prevent aphid escape. The plant choice of aphids was recorded after 1 h by counting the insects that climbed the arena and were settled beneath the leaf area exposed by the arena openings. The number of unresponsive aphids per replicate was also recorded and accounted for in the statistical model testing proportion of feeding choices (see below: “*Statical analysis*”). This feeding choice test was conducted between 9:00 and 17:00 in a greenhouse chamber with controlled abiotic conditions (22 ± 1 °C, 60 ± 10% RH, 16:8 h dark:light, 200 ± 20 μmol/s supplied by incandescent lights).

### Virus transmission assay

We examined the effects of endosymbionts on virus transmission when excluding all (previously tested) effects on aphid traits critical for disease spreading, such as behavioral preferences (e.g., increased preferences of virus-vectoring aphids for uninfected plants) and performance (e.g., increased population growth and dispersal rate of aphids on virus-infected plants). Therefore, this experimental design is inherently conservative for patterns of virus spreading. For this assay, groups of five wingless adults were placed on PEMV-infected plants for an acquisition access period of 48 h (per aphid line: three virus-infected source plants with five aphids in each). Subsequently, the aphids were individually transferred to a ten-day-old fava bean seedling and the system was sealed by the air permeable cellophane bag. The insects were allowed to feed on the seedlings for 48 h as virus inoculation access period, then the aphids and the cellophane bag were removed from the plants. Plants were kept the entirety of the experiment in the climate chamber and each individual seedling consisted of one experimental replicate (15 data points per aphid line). Given that virus loads can vary across genetically nonidentical plants and across tissues of the same host plants, we replicated this assay with a modified design to account for potential biased effects from low number of source plants for virus acquisition by aphids. Therefore, this latter experiment batch included a larger number of source plants for virus acquisition by the aphids, and a slightly larger sample size (per aphid line: ten virus-infected source plants with two aphids in each; 20 data points per aphid line). The data from both transmission assay batches were analyzed in combination, controlling for batch effects and variable number of aphids per source plants (pseudo replicate per replicate; see “*Statical analysis*”).

Seven days post virus inoculation, the leaves of every plant were collected in a paper bag, flash-frozen in liquid nitrogen and lyophilized for 48 h. Dried leaves were ground to powder using three 3 mm round glass beads in 50 ml falcon tubes in the Geno/grinder. From the homogenized powder, we sampled 0.1 ml in a 1.5 ml Eppendorf tube for RNA extraction using TRI Reagent (Sigma Aldrich) (details in supplementary information). Extracted RNA from leaf samples was treated with DNase I and reverse transcribed with RevertAid First Strand cDNA Synthesis Kit (Thermo Fisher Scientific) following manufacturer’s instructions. The qRT–PCR was performed in duplicates on a StepOnePlus Real-Time PCR System (Applied Biosystems) following PCR program recommended with the KAPA SYBR FAST qPCR mix (Sigma Aldrich, details in supplementary information; Table S4 for primers details). In addition, a melting curve was performed to verify the specificity of each PCR amplification. Cutoff points for Ct values (Cycle threshold, or Cycle of quantification) indicating positive PEMV infection was Ct ≤35.

### Statical analysis

All the statistical analyses were performed in R version 4.2.1 [74].

To analyze plant traits, a one-way non-parametric ANOVA was implemented to test the effects of virus infection on the biomass weight and hormone levels of plants (random effect: experimental block; R package ARTool [75]).

Plant and aphid metabolites were initially visualized with Discriminant Analysis of Principal Components (DAPC, R package adegenet [76]). Next, we center-scaled all the compounds before using them as response variables for a permutational analysis of variance (PERMANOVA) [77] based on Euclidean distances. PERMANOVA predictors were virus for the plant data set and the interaction of virus infection and endosymbionts for the aphid data set. Using these same predictors per data set, we tested differences in levels of chemical groups with generalized linear mixed models followed by multiple group comparisons with Tukey’s test (GLMM, distribution: gaussian with log link; random effect: experimental block; R package lm4 [78] and emmeans [79]). Subsequently, linear analysis, two-way non-parametric ANOVA and predictive models were employed to evaluate main effects of symbiotic bacteria and virus status on the overall metabolites of aphids (details in supplementary information). The compounds derived from these analyses were combined and their mean were represented in a heatmap to visualize treatments difference (R package superheat [80]).

We tested the effects of virus infection, endosymbionts, and their interactions on aphid population growth in the performance assay with GLMMs (distribution: poisson with log link; random effect: experimental block and day of count per replicate). In a subsequent model, we evaluated if removal of dispersed aphids influenced population count on the last day (day 20). For that, aphid counts were normalized by the starting population until the dispersal event, which generated population growth rates over time. Upon dispersal and removal of dispersed aphids from the assay, rates of growth were calculated based on the count of remaining aphid population multiplied by the growth rate prior to aphid removal. These population growth indices were then applied to population counts throughout the course of the experiment, and the new dataset was fitted with a GLMM as described above. We then compared estimates from both models, which indicated no effects of aphid removal on population growth until the last counting day in the experiment—day 20th— (Table S5). GLMMs were also used to determine the effects of endosymbionts, virus presence, and their interactions on the weight of aphids (distribution: gaussian with log link; random effect: experimental block), on rates of dispersal, and on host plant preferences of aphids in the dispersal and in the dual choice arena tests (distribution: binomial; weights for dispersal rate: population size, weights for host plant choices: number of responsive aphids per replicate; random effect: experimental block).

We tested the proportion of successful PEMV transmission by aphids harboring different symbionts using a GLMM followed by multiple group comparisons with Tukey’s test (GLMM, distribution: binomial with logit link; random effect: experimental batch and aphids per source plants; R package lm4 [78] and emmeans [79]).

### Supplementary information

Suplementary material

Supplementary table - List of metabolites in plant and aphid tissues

## Data Availability

All data generated or analyzed during this study are included in this published article (and its supplementary information files). Source data is available at ETH Zürich repository (10.3929/ethz-b-000630704).
