# Supplementary material for: Endosymbionts modulate virus effects on aphid-plant interactions
Source: ISME J. 2023 Nov 18;17(12):2441–51. doi: 10.1038/s41396-023-01549-z (PMC10689485; doi:10.1038/s41396-023-01549-z)
Supplement: Supplementary file 1 — Suplementary material [file 41396_2023_1549_MOESM1_ESM.pdf]

Supplementary Information

Endosymbionts modulate virus effects on aphid-plant interactions

Patricia Sanches[1], Consuelo M. De Moraes[1], Mark C. Mescher[1*]

[1]Department of Environmental Systems Science, ETH Zürich, Zürich, Switzerland

This file includes:

Extended Methods

Supplementary Figures S1-S7

Supplementary Tables S1-S5

Extended Methods

Phloem extraction

The top shoot of each plant was used for an EDTA-facilitated phloem extraction as described previously [1]. Briefly, the top stem was immersed in a 2 ml Eppendorf tube filled with 1.4 ml of 20 mM K_2-EDTA solution for 1 h. The stem was rinsed thoroughly with Milli-q water and subsequently immersed into in a 2 ml Eppendorf tube filled with 1.4 ml of autoclaved Milli-q water. The tubes were accommodated inside a humidified acrylic cage (30 cm width x 30 cm length x 30 cm height) under the lights in the climate chamber and phloem collection was carried through osmosis for 8 h. After this period, the Eppendorf tube was flash-frozen in liquid nitrogen and stored at -80 °C for further aqueous metabolites analysis.

Phytohormones extraction

Phytohormones were extracted by adding to the plant samples 650 ml of 100% methanol, 750 ml of 10 mM ammonium acetate and 50 ml of 1ng/ml isotope-labelled standards (d6-ABA, d5-JA, d4-SA, and d5-IAA). The tubes were vortex for 20 s, sonicated for 15 min and centrifuged for 10 min at 20,000 g at room temperature. Subsequently, 1 ml of the supernatant was transferred into a new 2 ml Eppendorf tube and dried in a speed-vacuum for 8 h. After this period, samples were resuspended in 100 ml of 0.1% formic acid and vortexed for 30 s. The tubes were incubated in ice for 10 min and then centrifuged at 20,000 g for 10 min at room temperature. Finally, samples were transferred into a 2 ml screw-cap glass vial with insert for chemical analysis.

Extraction of aqueous metabolites from plants and aphids

The metabolites of leaves and aphid samples were extracted following an adapted version of the protocol described previously[2]. We added to the samples 1.4 ml of 100% methanol and 6 µL of 2 mg/ml solution of Ribitol as internal standard. Samples were vortex for 10 s, shaken for 10 min at 70 °C in a thermomixer at 950 rpm and centrifuged for 10 min at 11000 g. The supernatant was transferred to a 4 ml labeled glass vial with 750 µl of chloroform and 1400 µl of dH2O at 4 °C. Samples were vortex for 10 s and centrifuged for 15 min at 2200 g. We transferred 150 µl of the upper polar phase into a 2 ml labeled Eppendorf tube and samples were completely dried in a speed-vacuum. Subsequently, the dried contents in the tubes were derivatized by adding 40 µl of 20 mg/ml of methoxyamination diluted in pure pyridine. Samples were shaken for 2h at 37 °C in the

thermomixer at 950 rpm. After this period, we added to the leaves samples 70 µl of MSTFA (N-Methyl-N-(trimethylsilyl)trifluoroacetamide)) and tubes were shaken once more for 2h at 37 ºC in the thermomixer at 950 rpm. To the aphid samples, we added 70 µl of MSTFA + 1% TMCS (trimethylchlorosilane) followed by the same incubation step in the thermomixer, as an updated version of the protocol to increase the resolution of recovered metabolites. Finally, samples were transferred into a screw-cap glass vial with insert for chemical analysis. The polar metabolites of phloem samples were extracted following a short version of this protocol. We added to the phloem samples 6 µL of 2 mg/ml solution of Ribitol as internal standard. The content in the tubes were dried completely in a speed-vacuum and the derivatization step was carried out as described for the leaves samples.

RNA isolation of leaves samples

RNA extraction from leaves samples were carried out as methods described previously[3]. Briefly, the total RNA of leaves was isolated by adding 1 ml of TRI Reagent (Sigma Aldrich) to the samples. Followed a 5 min of incubation at room temperature, we added 500 µl of chloroform into the tubes. Samples were vortex for 1 min and centrifuged at 18000 g for 5 min at 4 ºC. We transferred the upper phase into a new labeled tube and added to the samples 1 µl of glycogen, an aliquot (1:10 of the total water phase volume) of 3 M sodium acetate (pH 5.2) and 1 vol of isopropanol. Tubes were mixed by inverting multiple times and were incubated on ice for 30 min for RNA precipitation. Samples were centrifuged at 18000 g for 30 min at 4 ºC and the resulting supernatant was discarded. The RNA pelleted was washed twice with ice-cold 80% (vol/vol) ethanol, resuspended in DEPC-treated water, and then stored at −20 °C.

Variable selection process for aqueous metabolites

Predictive models were employed to evaluate main effects of endosymbionts and virus status on the overall metabolites of aphids. The variable selection process focused on selecting an optimal classification model with high sensitivity and accuracy, while identifying the minimal number of compounds needed for effective prediction. The metabolite data set was partitioned into a training set and a test set using 80% and 20% of the data, respectively. Using the training set, we trained five machine learning classification algorithms—random forest (rf)[4], adaptive boosting (adaboost)[5], extreme gradient boosting (xgbDART)[6], support vector machines with radial basis function Kernel (svmRadial)[7], and multivariate adaptive regression spline (earth)[8] (function caretList, resampling parameters: 10-fold cross-validation with 3 repeats; R package caretEnsemble[9]). We used a

regression stack with these algorithms to improve prediction accuracy when individual model's accuracy was below 0.7 (caretStack, method: glm, metric: accuracy, resampling parameters: 10-fold cross-validation with 3 repeats; R package caretEnsemble[9]). A list of compounds important for treatment predictions were generated from individual algorithms (function VarImp; R package caret[10]). In addition to the predictive models, we employed liner analysis (t-tests, P value < 0.05 and log2 fold change > 0.5, function lmFit; R package limma[11]) and two-way non-parametric ANOVAs (random effect: experimental block; R package ARTool[12,13]) to complement the identification of differentially expressed compounds between the treatments. The compounds derived from all these analysis were combined and their mean values per treatment were represented in a heatmap (R package superheat[14]).

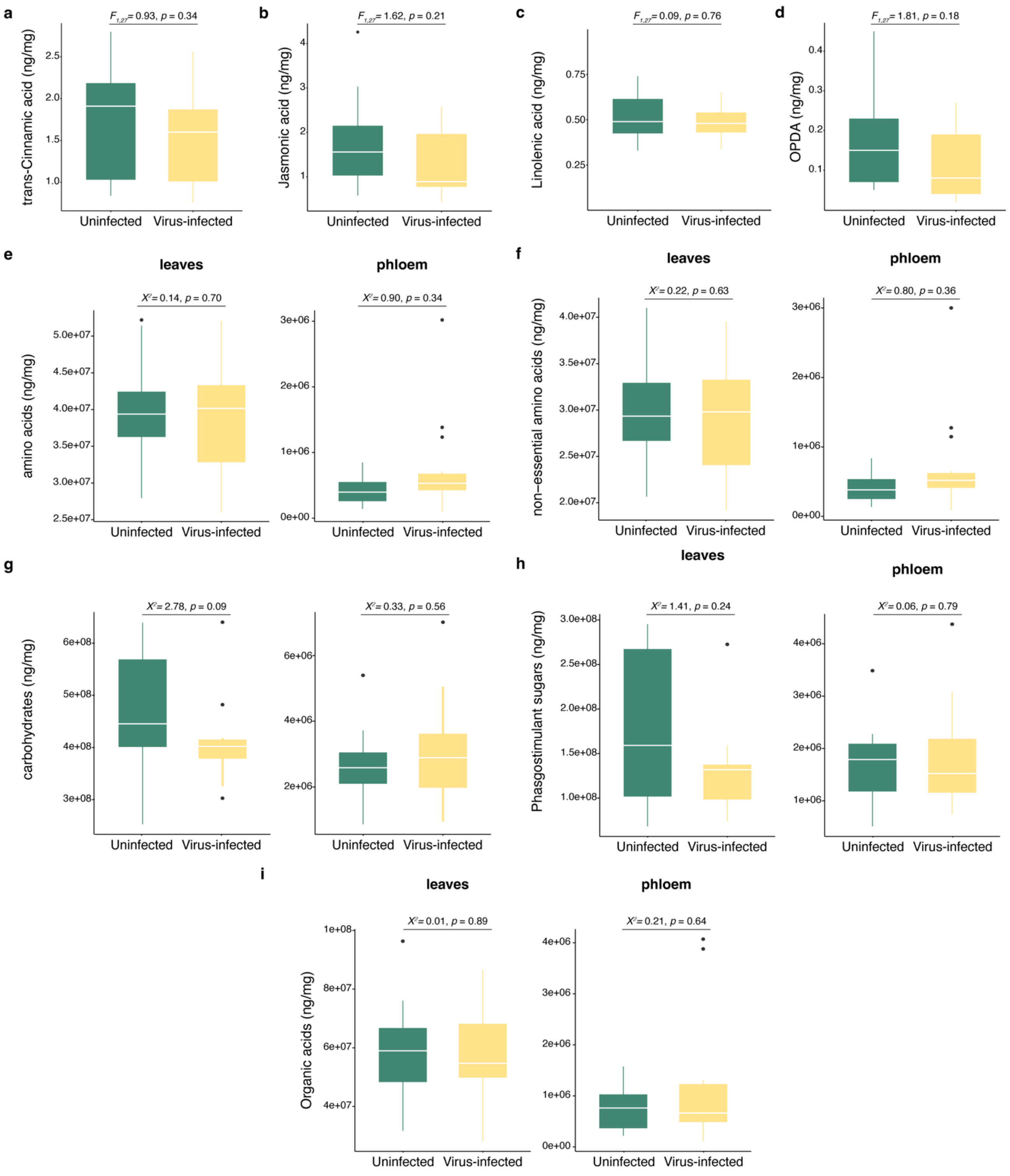

Fig. S1: Phytohormones and metabolite groups with similar levels in uninfected and plants infected with pea enation mosaic virus. a Levels of trans-Cinnamic acid, **b** Jasmonic acid, **c** Linolenic acid, and **d** 12-oxophytodienoic acid (OPDA) of uninfected (green) and virus-infected fava beans (yellow); one-sided nonparametric ANOVA, aligned rank transform. **e** Levels of total amino acids, **f** non-essential amino acids, **g** carbohydrates, **h** phagostimulant sugars, and **i** organic acids in the leaves and phloem of uninfected (green) and virus-infected fava beans (yellow); generalized linear mixed models (boxplots display median line, interquartile range (IQR) boxes, 1.5 × IQR whiskers; point indicates outlier observations; $N = 14$).

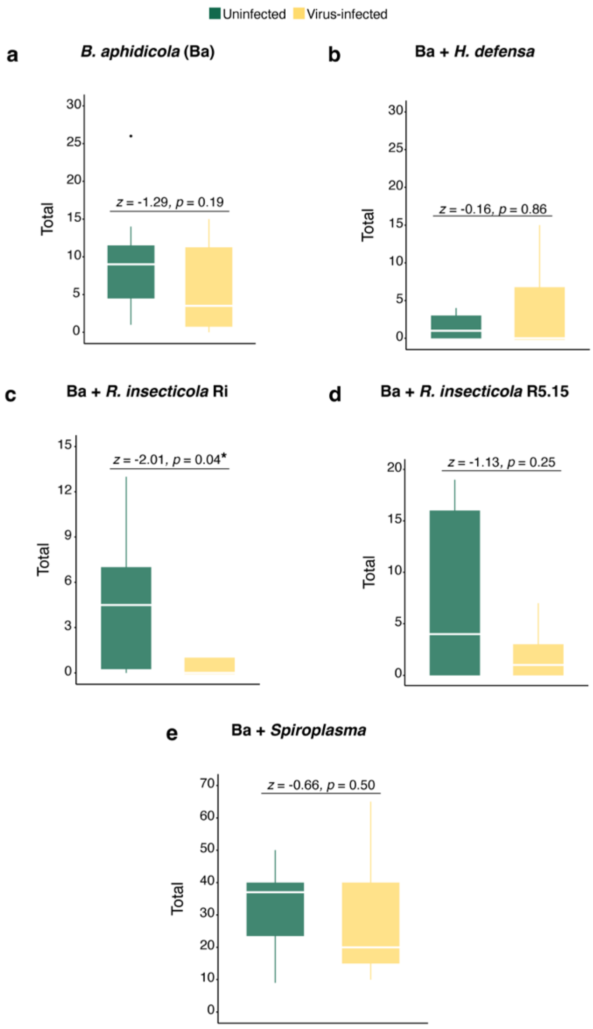

Fig. S2: Total of winged morphs in aphid populations harboring different endosymbionts and growing on uninfected or virus-infected plants. a-e Number of winged morphs after 20 days of population growth on uninfected (green) or plants infected with pea enation mosaic virus (PEMV; yellow). Aphids differed in endosymbiont composition: only *B. aphidicola* (Ba) (**a:** uninfected $N = 7$, virus-infected $N = 8$), Ba + *H. defensa* (**b:** uninfected $N = 9$, virus-infected $N = 9$), Ba + *R. insecticola* strain Ri (**c:** uninfected $N = 9$, virus-infected $N = 9$), Ba + *R. insecticola* strain R5.15 (**d:** uninfected $N = 10$, virus-infected $N = 8$), and Ba + *Spiroplasma* (**e:** uninfected $N = 7$, virus-infected $N = 8$). Generalized linear mixed model (interaction of endosymbionts and virus status: $X^2 = 2.51$, $p > 0.05$; virus status: $X^2 = 3.69$, $p = 0.05$; symbiont: $X^2 = 93.67$, $p < 0.0001$; boxplots display median line, interquartile range (IQR) boxes, $1.5 \times$ IQR whiskers; panels display comparisons within each aphid line according to generalized linear mixed models, *$p < 0.05$).

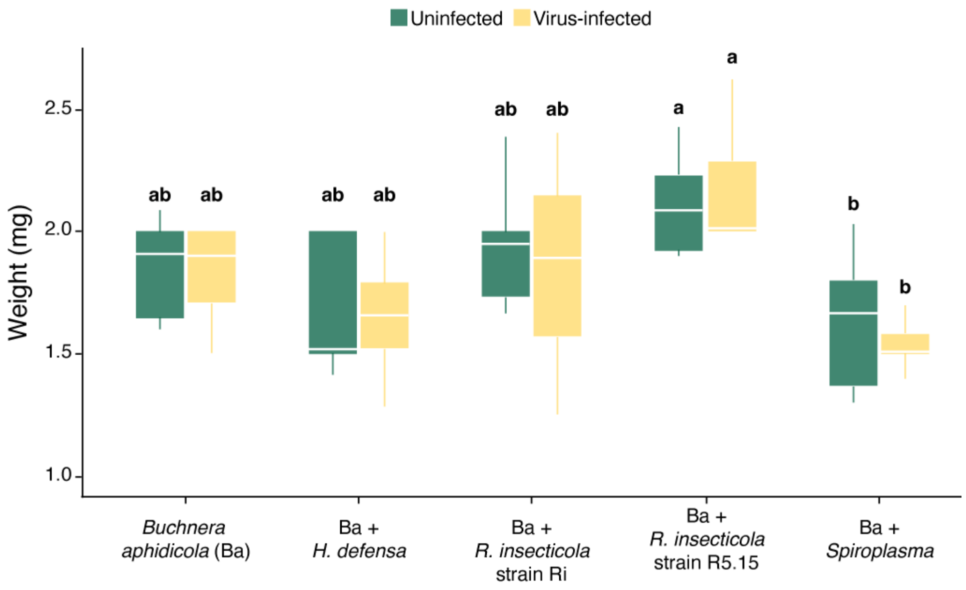

Fig. S3: Weight of winged aphids harboring different endosymbionts and growing on uninfected or virus-infected plants. Aphids grew on uninfected (green) or plants infected with pea enation mosaic virus (PEMV; yellow) and differed in endosymbiont composition: only *B. aphidicola* (Ba) (uninfected $N = 7$, virus-infected $N = 8$), Ba + *H. defensa* (uninfected $N = 9$, virus-infected $N = 9$), Ba + *R. insecticola* strain Ri (uninfected $N = 9$, virus-infected $N = 9$), Ba + *R. insecticola* strain R5.15 (uninfected $N = 10$, virus-infected $N = 8$), and Ba + *Spiroplasma* (uninfected $N = 7$, virus-infected $N = 8$). Generalized linear mixed model followed by Tukey's multiple comparisons (interaction of endosymbionts and virus status: $X^2 = 0.73$, $p > 0.05$; virus status: $X^2 = 0.43$, $p > 0.05$; symbiont: $X^2 = 11.01$, $p = 0.02$; same letters indicate no statistical difference; boxplots display median line, interquartile range (IQR) boxes, 1.5 × IQR whiskers).

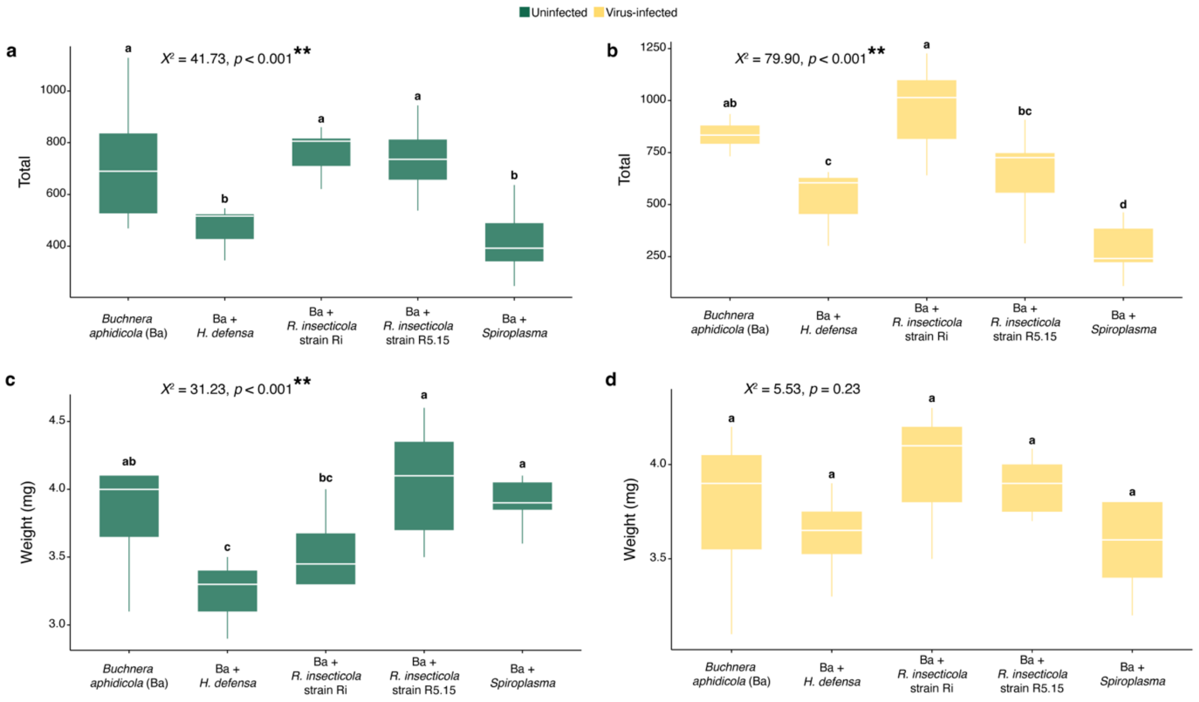

Fig. S4: Effects of endosymbionts on the performance of wingless aphids growing on either uninfected or virus-infected plants. a Total of wingless population growing on uninfected (green) **b** and on plants infected with pea enation mosaic virus (PEMV; yellow). **c** Average biomass of wingless aphids growing on uninfected (green) and **d** on PEMV-infected plants (yellow). Aphids differed in endosymbiont composition: only *B. aphidicola* (Ba) (N = 7), Ba + *H. defensa* (N = 9), Ba + *R. insecticola* strain Ri (N = 9), Ba + *R. insecticola* strain R5.15 (N = 10), and Ba + *Spiroplasma* (N = 7). Generalized linear mixed models followed by Tukey's multiple comparisons (same letters indicate no statistical difference; boxplots display median line, interquartile range (IQR) boxes, 1.5 × IQR whiskers).

a

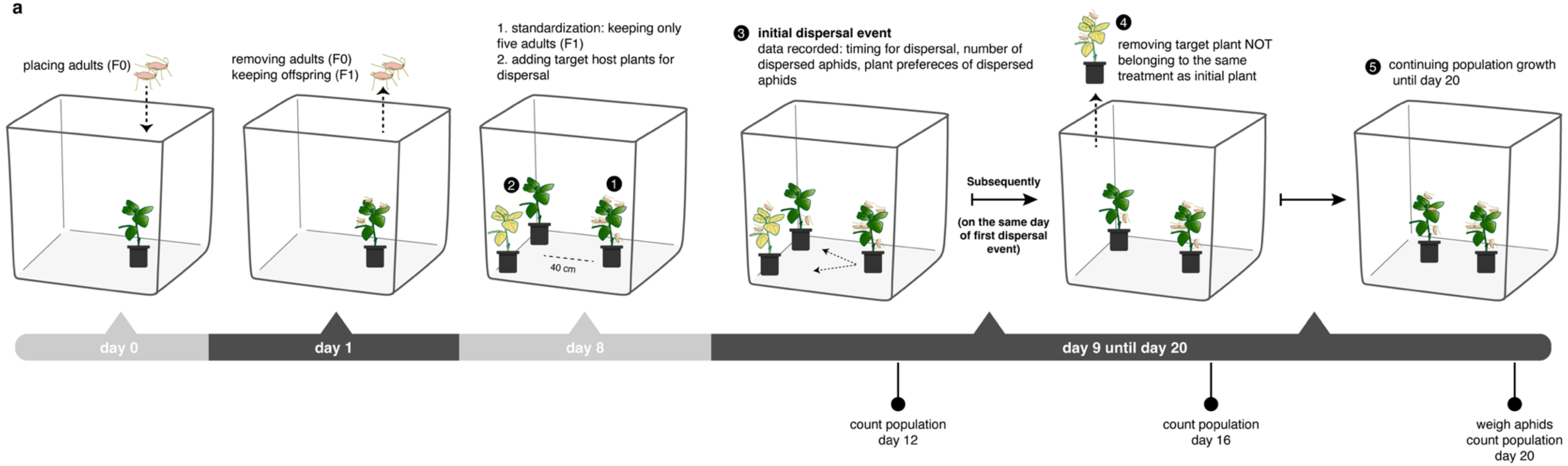

b

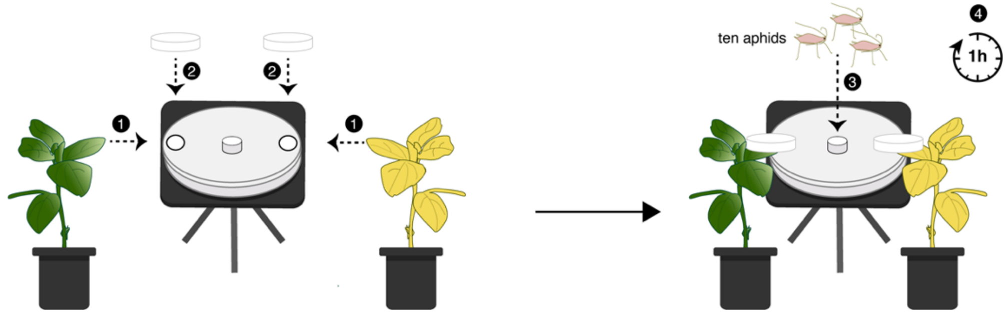

Fig. S5: Design of assays investigating aphid performance on and preferences for uninfected and virus-infected host plants. a Combined experiments assessing performance and initial dispersal event of aphids (numbers inside black dots indicate order of experimental step). For simplicity, the illustration represents the timeline of experiment steps using as a model uninfected plant as initial treatment for population growth and aphid emigration; however, the experiment was simultaneously carried out with virus-infected host plant as initial treatment. **b** Dual-choice arenas used for testing the feeding preferences of aphids (numbers inside black dots indicate order of experimental step: 1) placement of leaves onto arena; 2) placement of cotton pad onto leaves; 3) release of ten aphids into the arena; 4) total time for aphid choice test). In both illustrations, green plant indicates uninfected fava bean while yellow plant represents fava bean infected with pea enation mosaic virus.

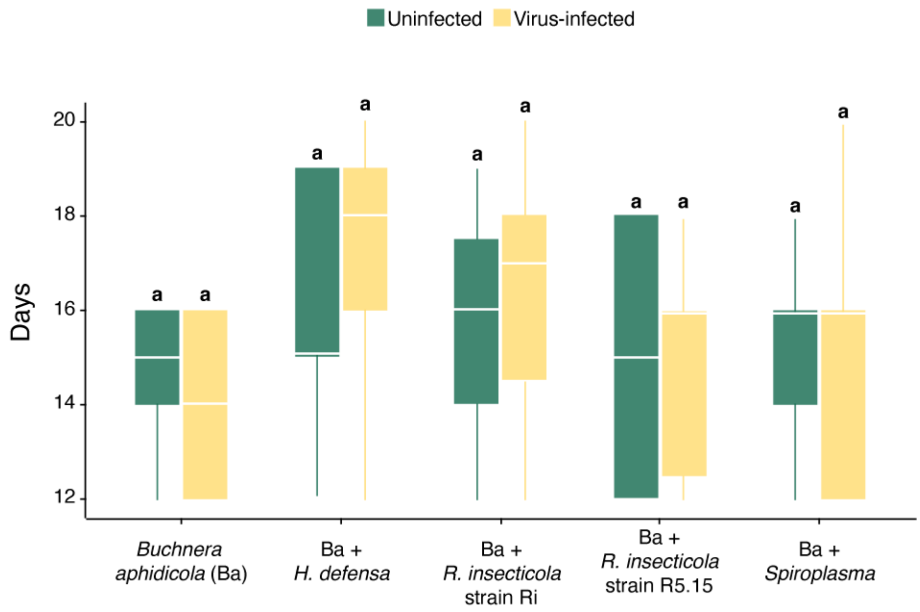

Fig. S6: Timing of first dispersion event of aphids harboring different endosymbionts and growing on uninfected or virus-infected plants. Days until the initial aphid dispersal from uninfected (green) or plants infected with pea enation mosaic virus (PEMV; yellow). Aphids differed in endosymbiont composition (bottom labels): only *B. aphidicola* (Ba) (uninfected $N = 7$, virus-infected $N = 8$), Ba + *H. defensa* (uninfected $N = 9$, virus-infected $N = 9$), Ba + *R. insecticola* strain Ri (uninfected $N = 9$, virus-infected $N = 9$), Ba + *R. insecticola* strain R5.15 (uninfected $N = 10$, virus-infected $N = 8$), and Ba + *Spiroplasma* (uninfected $N = 7$, virus-infected $N = 8$). Generalized linear mixed model (interaction of endosymbionts and virus status: $X^2 = 0.28$; $p > 0.05$; symbiont: $X^2 = 0.56$; virus status: $X^2 = 0.94$; same letter indicates no statistical difference; boxplots display median line, interquartile range (IQR) boxes, 1.5 × IQR whiskers).

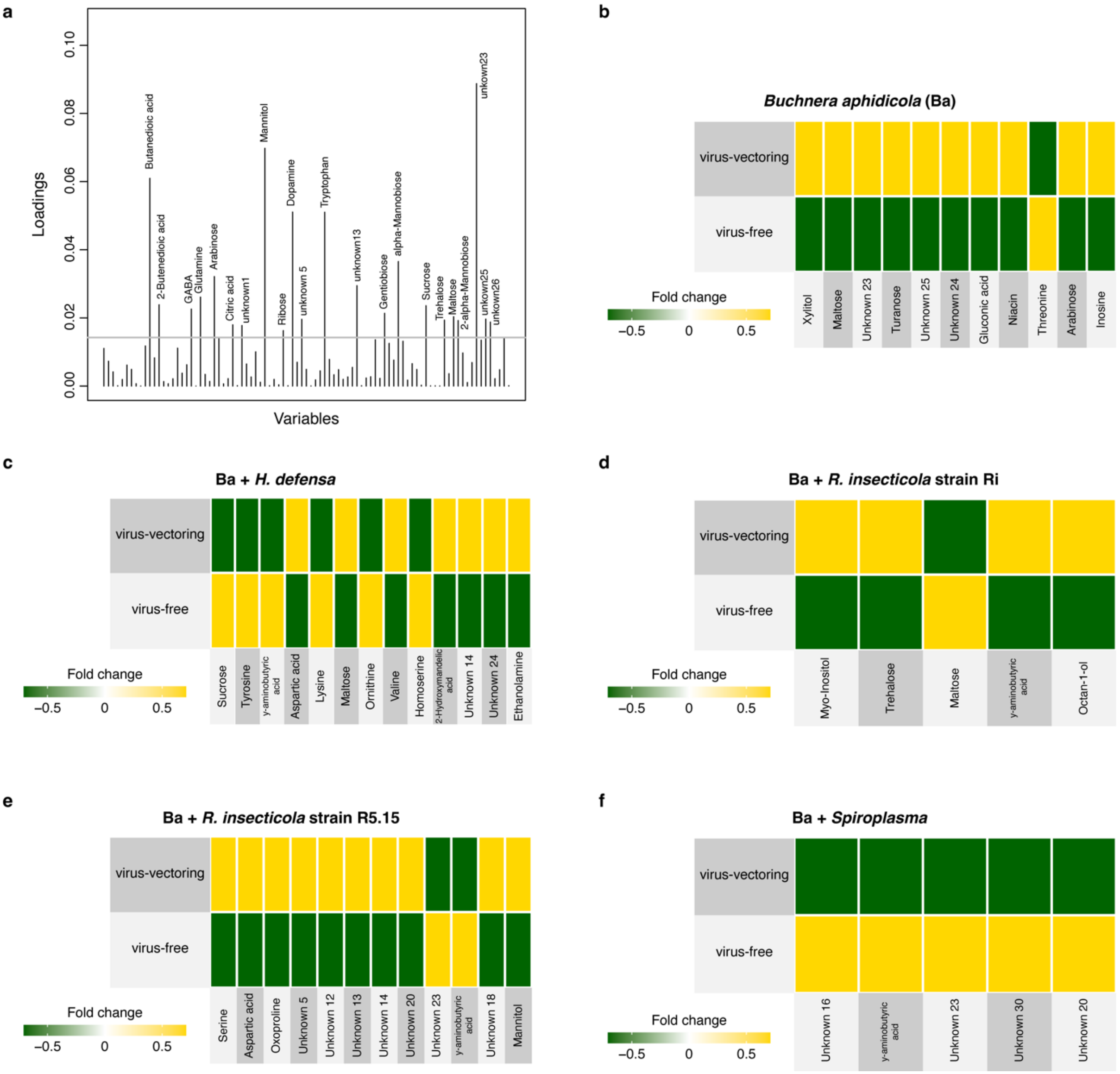

Fig. S7: Key metabolites distinguishing virus-vectoring from virus-free aphids harboring different endosymbionts. **a** Variables contributing to discriminant analysis of principal components (DAPC). **b-f** Heatmaps representing the fold change of compounds important for discriminating virus-vectoring from virus-free aphids within each aphid line according to predictive models (individual or stacked classification algorithms: random forest, adaptive boosting, extreme gradient boosting, support vector machines with radial basis function Kernel, and multivariate adaptive regression spline; resampling parameters: 10-fold cross-validation with 3 repeats; model accuracy ≥ 0.70) and according to one-sided nonparametric ANOVAs ($p \leq 0.05$). Virus-vectoring and virus-free aphids differed on endosymbiont sets: only *B. aphidicola* (Ba) (**b**), Ba + *H. defensa* (**c**), Ba + *R. insecticola* strain Ri (**d**), Ba + *R. insecticola* strain R5.15 (**e**), and Ba + *Spiroplasma* (**f**).

Table S1. Effects of virus infection on levels of individual amino acids detected in plant tissues.

Levels of amino acids in the leaves and phloem of uninfected and plants infected with pea enation mosaic virus (PEMV) (N = 14) followed by statistical comparisons using log rank-transform ANOVAs (see Fig. 1)

Amino acids	Leaves				Phloem			
	Uninfected (mean ± SE)	Virus-infected (mean ± SE)	$F_{1,27}$	p	Uninfected (mean ± SE)	Virus-infected (mean ± SE)	$F_{1,27}$	p
Valine*	not detected	not detected			2.77E+03 ± 5.07E+02	7.32E+03 ± 2.17E+03	1.62	0.21
Alanine	6.95E+05 ± 4.30E+04	8.08E+05 ± 5.03E+04	2.01	0.16	5.82E+03 ± 1.09E+03	5.01E+03 ± 1.09E+03	0.66	0.42
Leucine*	not detected	not detected			1.69E+03 ± 2.65E+02	4.78E+03 ± 1.55E+03	2.04	0.16
Isoleucine*	4.76E+06 ± 1.28E+05	4.34E+06 ± 2.33E+05	1.75	0.19	597.85 ± 48.01	789.06 ± 113.55	1.89	0.18
Serine	6.06E+06 ± 9.03E+05	5.75E+06 ± 6.47E+05	0.01	0.95	3.39E+04 ± 9.11E+03	8.61E+04 ± 4.16E+04	0.94	0.34
Threonine*	1.42E+06 ± 1.67E+05	1.55E+06 ± 1.21E+05	0.51	0.48	7.22E+03 ± 1.28E+03	1.78E+04 ± 5.04E+03	2.20	0.15
Glycine	1.12E+07 ± 2.99E+05	1.08E+07 ± 3.52E+05	0.63	0.43	2.32E+04 ± 2.38E+03	3.40E+04 ± 5.10E+03	4.53	**0.04**
Asparagine	1.19E+06 ± 8.44E+04	1.09E+06 ± 8.62E+04	0.64	0.42	not detected	not detected		
Ethanolamine	9.94E+05 ± 5.69E+04	7.50E+05 ± 4.53E+04	16.26	**<0.001**	1.62E+03 ± 3.85E+02	4.94E+03 ± 2.61E+03	0.38	0.54
Aspartic acid	7.64E+05 ± 7.70E+04	1.07E+06 ± 1.37E+05	2.85	0.10	3.29E+03 ± 1.04E+03	7.19E+03 ± 1.94E+03	1.25	0.27
Oxoproline	5.19E+06 ± 3.06E+05	5.36E+06 ± 3.76E+05	0.22	0.64	not detected	not detected		
y-aminobutyric acid	1.76E+06 ± 1.21E+05	1.97E+06 ± 2.10E+05	0.65	0.42	9.23E+04 ± 2.46E+04	2.44E+05 ± 9.45E+04	0.93	0.34
Phenylalanine*	3.30E+06 ± 2.44E+05	3.65E+06 ± 2.21E+05	0.37	0.54	not detected	not detected		
Glutamic acid	not detected	not detected			2.97E+04 ± 6.05E+03	9.82E+04 ± 4.87E+04	1.12	0.29
Ornithine	not detected	not detected			494.90 ± 182.74	855.90 ± 301.88	1.08	0.30
Tyrosine	2.38E+06 ± 2.94E+05	1.66E+06 ± 2.00E+05	3.92	0.05	2.35E+05 ± 2.81E+04	2.25E+05 ± 3.56E+04	0.12	0.72

* indicates essential amino acid

Table S2. Effects of endosymbionts on groups of metabolites in aphids.

Statistical results of log rank-transform ANOVAs testing the levels of groups of metabolites in aphids harboring different endosymbionts (see Fig. 4).

Response variables	Endosymbionts		*Post-hoc comparisons among aphid lines				
	$F_{4,80}$	p	*B.aphidicola* (Ba)	(Ba) + *H. defensa*	(Ba) + *R. insecticola* Ri	(Ba) *R. insecticola* R5.15	(Ba) + *Spiroplasma*
Amino acids	2.23	0.07	a	a	a	a	a
Carbohydrates	8.14	**<0.001**	b	b	b	b	a
Organic acids	5.94	**<0.001**	a	a	a	a	b
Pyrimidines	3.15	**0.01**	a	a	ab	ab	b
Purines	0.77	0.54	a	a	a	a	a
Fatty acyls	3.05	**0.02**	ab	b	b	ab	a
gamma-aminobutyric acid (GABA)	1.70	0.15	a	a	a	a	a

* Same letters do not differ according to Tukey's pairwise comparisons.

Table S3. Summary of endosymbionts effects on aphid phenotypes in ways that appear conducive with plant virus transmission. Outcome of comparisons between virus-vectoring and virus-free aphids within each aphid line. Traits considered to be conducive with transmission of pea enation mosaic virus (PEMV) are the following: enhanced performance, patterns of behavior conducive with virus acquisition or subsequent dissemination, and rates of virus transmission above 70%.

**Assay**	**Trait**	**Obligate symbiont**	**Facultative symbiont**			
		B. aphidicola (Ba)	Ba + *H. defensa*	Ba + *R. insecticola* Ri	Ba + *R. insecticola* R5.15	Ba + *Spiroplasma*
Aphid performance	Wingless population (Fig. 2)	0	0	+	0	-
	Wingless weight (Fig. 2)	0	+	+	0	-
	Winged production (Fig. S2)	0	0	-	0	0
	Winged weight (Fig. S3)	0	0	0	0	0
Aphid behavior: dispersal assay	Dispersal rate (Fig. 3 a-e)	+	0	0	0	+
	Virus effects on behavioral patterns (Fig. 3 f-j:(*i*))	0	0	+	0	0
	Plant preferences virus-free aphids (Fig. 3 f-j:(*ii*))	0	0	0	0	0
	Plant preferences virus-vectoring aphids (Fig. 3 f-j:(*iii*))	0	0	+	0	0
Aphid behavior: test arena	Virus effects on behavioral patterns (Fig. 3 k-o:(*i*))	0	+	0	-	+
	Plant preferences virus-free aphids (Fig. 3 k-o:(*ii*))	0	+	0	0	0
	Plant preferences virus-vectoring aphids (Fig. 3 k-o:(*iii*))	0	0	0	0	0
Virus transmission	Rates of PEMV transmission (Fig. 5)	31%	77%	37%	57%	60%

"+" and transmission rate highlighted in red indicate positive effects on PEMV transmission.
"0" represents neutral effects on PEMV transmission.
"-" denotes negative effects on PEMV transmission.

Table S4. Thermocycler conditions for diagnostics of aphid endosymbionts and pea enation mosaic virus.

Target	Forward primer		Reverse primer		Amplicon size	Program
Buchnera aphidicola	16SA1 [15]	AGAGTTTGATCMTGGCTCAG	Buch16S1R [15]	CTTCTGCGGGTAACGTCACGAA	492 bp	A
Hamiltonella defensa	10F [16]	AGTTTGATCATGGCTCAGATTG	T419/TO419 [17]	AAATGGTATTCGCATTTATCG	471 and 305 bp	A
Regiella insecticola	10F [16]	AGTTTGATCATGGCTCAGATTG	U443R [17]	GGTAACGTCAATCGATAAGCA	414 and 480 bp	A
Spiroplasma	ApDnaAF1 [15]	ATTCTTCAGTAAAAATGCTTGGA	ApDnaAR1[15]	ACACATTTACTTCATGCTATTGA	445 bp	A
	16SA1 [15]	AGAGTTTGATCMTGGCTCAG	TKSSsp [15]	TAGCCGTGGCTTTCTGGTAA	400-500 bp	
Pea enation mosaic virus	PEMV-1 [18]	GCAATCCTACAGGACCTTCATA	PEMV-1 [18]	CTCATCGTCTTCCGTGTCATC	121 bp	B
Vicia faba (Housekeeping gene)	CYP2 [19]	TGCCGATGTCACTCCCAGAA	CYP2 [19]	CAGCGAACTTGGAACCGTAGA	120-200 bp	B

Cycling conditions

Program A - PCR: 95°C 2min, 10 cycles of (95°C 30 s, 65°C→56°C in 1°C steps each cycle 30s, 72°C 1min), 25 cycles of (95°C 30s, 55°C 30s, 72°C 1min), 72°C 6min

Program B - qPCR: 95°C 3 min, 40 cycles of (95°C 15 s, 60°C 30s, 72°C 30s), 72°C 6min. For melting curve analysis, a dissociation step cycle was added (55°C 10 s, 0.5°C for 10 s until 95°C)

Table S5. Aphid population growth accounting or not for removal of dispersed aphids.

Statistical output of generalized linear mixed models testing effects of symbionts and plant virus presence on total of wingless aphids after 20 days using non-normalized or normalized dataset by aphid removal after dispersal event.

	**Model with non-normalized population count**			**Model with normalized population count**		
Effects of symbiont and virus status on aphid population growth (full model):						
Predictors	Chisq	Df	Pr(>Chisq)	Chisq	Df	Pr(>Chisq)
Symbiont	140.418	4	<2.2e-16	136.433	4	<2.2e-16
Virus_status	0.536	1	0.464	0.154	1	0.694
Interaction: Symbiont*Virus_status	15.891	4	0.003	14.288	4	0.006
Pairwise comparisons within the same aphid line growing on uninfected vs virus-infected plants:						
Symbiont	Estimate ± SE	z.ratio	p.value	Estimate ± SE	z.ratio	p.value
only *B. aphidicola* (Ba)	0.826 ± 0.090	-1.748	0.080	0.850 ± 0.093	-1.484	0.137
Ba + *H. defensa*	0.973 ± 0.117	-0.226	0.821	0.996 ± 0.120	-0.033	0.973
Ba + *R. insecticola* strain Ri	0.817 ± 0.072	-2.274	0.023	0.833 ± 0.074	-2.032	0.042
Ba + *R. insecticola* strain R5.15	1.144 ± 0.119	1.281	0.200	1.149 ± 0.121	1.310	0.190
Ba + *Spiroplasma*	1.490 ± 0.232	2.553	0.010	1.487 ± 0.233	2.529	0.011

References

1. Tetyuk O, Benning UF, Hoffmann-Benning S. Collection and analysis of Arabidopsis phloem exudates using the EDTA-facilitated method. *J Vis Exp. 2013;* 80:51111.
2. Lisec J, Schauer N, Kopka J, Willmitzer L, Fernie AR. Gas chromatography mass spectrometry–based metabolite profiling in plants. *Nat Protoc.* 2006; 1:387–396.
3. Devers EA, Brosnan CA, Sarazin A, Albertini D, Amsler AC, Brioudes F, Jullien PE, Lim P, Schott G, Voinnet O. Movement and differential consumption of short interfering RNA duplexes underlie mobile RNA interference. *Nat Plants*. 2020; 7:789-99.
4. Liaw A, Wiener M. Classification and regression by randomForest. *R news*. 2002;18-22.
5. Alfaro E, Gamez M, Garcia N. adabag: An R package for classification with boosting and bagging. *J Stat Soft*. 2013;54:1-35.
6. Chen T, He T, Benesty M, Khotilovich V, Tang Y, Cho H, Chen K, Mitchell R, Cano I, Zhou T. Xgboost: extreme gradient boosting. *R package version.* 2015; 1:1–4 (2015).
7. Karatzoglou A, Hornik K, Smola A, Zeileis A. Kernlab - an S4 package for Kernel methods in R. *J Stat Soft*. 2004; 11: 9.
8. Milborrow S, Hastie T, Tibshirani R. earth: multivariate adaptive regression splines. *R package version* 5. 2017.
9. Deane-Mayer ZA, Knowles JE. caretEnsemble: ensembles of caret models. *R package version* 2. 2016.
10. Kuhn M. Building predictive models in R using the caret package. *J Stat Soft*. 2008; 28:1–26.
11. Ritchie ME, Phipson B, Wu DI, Hu Y, Law CW, Shi W, Smyth GK. limma powers differential expression analyses for RNA-sequencing and microarray studies. *Nucleic Acids Res*. 2015; e47.
12. Elkin LA, Kay M, Higgins JJ, Wobbrock JO. An aligned rank transform procedure for multifactor contrast tests. *In the 34th annual ACM symposium on user interface software and technology*. 2021; 754–768.
13. Wobbrock JO, Findlater L, Gergle D, Higgins JJ. The aligned rank transform for nonparametric factorial analyses using only anova procedures. In *Proceedings of the SIGCHI conference on human factors in computing systems.* 2011; 143-146.

14. Barter RL, Yu B. Superheat: An R package for creating beautiful and extendable heatmaps for visualizing complex data. *J Comput Graph Stat.* 2018; 27: 910–922.
15. Fukatsu T, Tsuchida T, Nikoh N, Koga R. *Spiroplasma* symbiont of the pea aphid, *Acyrthosiphon pisum* (Insecta: Homoptera). *Appl Environ Microbiol.* 2001; 284–1291.
16. Sandström JP, Russell JA, White JP, Moran, NA. Independent origins and horizontal transfer of bacterial symbionts of aphids. *Mol Ecol.* 2001; 217–228.
17. Ferrari J, West JA, Via S, Godfray HCJ. Population genetic structure and secondary symbionts in host-associated populations of the pea aphid complex. *Evolution.* 2012; 375–390.
18. Lee BW, Basu S, Bera S, Casteel CL, Crowder DW. Responses to predation risk cues and alarm pheromones affect plant virus transmission by an aphid vector. *Oecologia*. 2021; 1005–1015.
19. Gutierrez N, Giménez MJ, Palomino C, Avila CM. Assessment of candidate reference genes for expression studies in *Vicia faba* L. by real-time quantitative PCR. *Mol Breed.* 2011; 13–24.
