# Supplementary material for: Endosymbionts modulate virus effects on aphid-plant interactions
Source: ISME J. 2023 Nov 18;17(12):2441–51. doi: 10.1038/s41396-023-01549-z (PMC10689485; doi:10.1038/s41396-023-01549-z)
Supplement: Supplementary file 2 — Supplementary table - List of metabolites in plant and aphid tissues [file 41396_2023_1549_MOESM2_ESM.pdf]

Sample type	Compound name	Simplified compound name	Confirmation with derivatized standard	Retention time (minutes)	Kovats Index
aphids	L-Alanine, TMS derivative	Alanine, TMS	yes	9.55	1003.16
aphids	Lactic Acid, 2TMS derivative	Latic acid, 2TMS	no	12.40	1093.51
aphids	L-Alanine, N-(trifluoroacetyl)-, trimethylsilyl ester	Alanine, NTFA-TMS	yes	12.86	1108.08
phloem, aphids	L-Valine, TMS derivative	Valine, TMS	yes	13.06	1114.24
leaves, phloem, aphids	L-Alanine, 2TMS derivative	Alanine, 2TMS	yes	13.48	1127.60
phloem	Oxalic acid, 2TMS derivative	Oxalic acid, 2TMS	no	14.41	1156.59
phloem, aphids	L-Leucine, TMS derivative	Leucine, TMS	yes	14.99	1174.64
leaves, phloem, aphids	L-Isoleucine, TMS derivative	Isoleucine, TMS	yes	15.60	1193.75
aphids	L-Valine, 2TMS derivative	Valine, 2TMS	yes	16.68	1231.01
leaves, phloem, aphids	L-Serine, 2TMS derivative	Serine, 2TMS	yes	17.81	1271.10
aphids	Ethanolamine, 3TMS derivative	Ethanolamine , 3TMS	yes	17.99	1277.26
aphids	L-Leucine, 2TMS derivative	Leucine, 2TMS	yes	18.19	1284.31
leaves, phloem	Phosphoric acid, 3TMS derivative	Phosphoric acid, 3 TMS	yes	18.30	1288.03
aphids	Niacin, TMS derivative	Niacin, TMS	no	18.73	1303.49
leaves, phloem, aphids	L-Threonine, 2TMS derivative	Threonine, 2TMS	yes	18.80	1306.04
leaves	L-Isoleucine, 2TMS derivative	Isoleucine, 2TMS	yes	18.96	1311.89

Sample Type	Compound name	Simplified compound name	Confirmation with derivatized standard	Retention time (minutes)	Kovats Index
leaves, phloem, aphids	Glycine, 3TMS derivative	Glycine, 3TMS	yes	19.06	1315.85
phloem, aphids	Butanedioic acid, 2TMS derivative	Butanedioic acid, 2TMS	no	19.20	1321.19
aphids	Uracil, 2TMS derivative	Uracil, 2TMS	no	19.93	1348.81
leaves, phloem	Glyceric acid, 3TMS derivative	Glyceric acid, 3TMS	no	19.98	1350.53
aphids	2-Butenedioic acid, (E)-, 2TMS derivative	2-Butenedioic acid, 2TMS	no	20.09	1354.71
aphids	L-Homoserine, 2TMS derivative	Homoserine, 2TMS	no	20.45	1368.17
leaves, phloem, aphids	Serine, 3TMS derivative	Serine, 3TMS	yes	20.59	1373.59
leaves	Asparigne, 3TMS	Asparigine, 3TMS	no	21.01	1389.43
leaves, phloem, aphids	L-Threonine, 3TMS derivative	Threonine, 3TMS	yes	21.20	1396.60
leaves, phloem, aphids	Ethanolamine, 3TMS derivative	Ethanolamine, 3TMS	yes	21.35	1402.45
aphids	L-Methionine, TMS derivative	Methionine, TMS	yes	21.60	1412.65
leaves, phloem, aphids	L-Aspartic acid, 2TMS	Aspartic acid, 2TMS	yes	22.18	1436.33
aphids	Homoserine, 3TMS derivative	Homoserine, 3TMS	yes	22.82	1462.33
aphids	3-Aminoisobutyric acid, 3TMS derivative	3-Aminoisobutyric acid, 3TMS	no	22.94	1467.18
aphids	L-Methionine, 2TMS derivative	Methionine, 2TMS	yes	23.30	1482.04

Sample Type	Compound name	Simplified compound name	Confirmation with derivatized standard	Retention time (minutes)	Kovats Index
leaves, phloem, aphids	Malic acid, 3TMS derivative	Malic acid, 3TMS	yes	23.75	1500.40
leaves, aphids	L-5-Oxoproline, 2TMS derivative	5-Oxoproline, 2TMS	no	24.50	1532.47
leaves, phloem, aphids	y-aminobutyric acid, 3TMS derivative	y-aminobutyric acid, 3TMS	yes	24.60	1536.91
aphids	DL-Phenylalanine, TMS derivative	Phenylalanine, TMS	yes	25.20	1562.68
aphids	L-Glutamine, 3TMS derivative	Glutamine, 3TMS	yes	25.34	1568.86
aphids	L-Threonic acid, tris(trimethylsilyl) ether, trimethylsilyl ester	Threonic acid	no	25.64	1581.47
leaves	Xylose, 4TMS derivative	Xylose, 4TMS	no	26.32	1611.29
leaves, phloem and aphids	L-Glutamic acid, 3TMS derivative	Glutamic acid, 3TMS	yes	26.75	1630.99
aphids	L-Phenylalanine, 2TMS derivative	Phenylalanine, 2TMS	yes	26.91	1638.10
phloem, aphids	D-Arabinose, tetrakis(trimethylsilyl) ether, ethyloxime (isomer 2)	Arabinose	no	28.35	1703.98
leaves, phloem, aphids	Xylitol, 5TMS derivative	Xylitol, 5TMS	no	29.09	1739.30
aphids	DL-Ornithine, 3TMS derivative	Ornithine, 3TMS	no	29.74	1770.43
aphids	Phosphoric acid, bis(trimethylsilyl) 2,3-bis[(trimethylsilyl)oxy]propyl ester	Phosphoric acid	no	30.16	1790.48
phloem, aphids	L-Ornithine, 4TMS derivative	Ornithine, 4TMS	no	31.00	1846.35
leaves, phloem, aphids	Citric acid, 4TMS derivative	Citric acid, 4TMS	no	31.29	1867.93
aphids	N-.alpha.-Acetyl-L-Lysine	Lysine	yes	31.72	1867.93

Sample Type	Compound name	Simplified compound name	Confirmation with derivatized standard	Retention time (minutes)	Kovats Index
aphids	unknown 1	unknown 1	no	31.82	1872.93
leaves	unknown 2	unknown 2	no	31.89	1876.29
phloem	unknown 3	unknown 3	no	31.91	1877.71
aphids	Adenine, 2TMS derivative	Adenine, 2TMS	no	32.03	1883.26
aphids	Tyrosine, 2TMS derivative	Tyrosine, 2TMS	yes	32.46	1905.42
leaves, phloem, aphids	D-Fructose, 1,3,4,5,6-pentakis-O-(trimethylsilyl)-, O-methyloxime	Fructose	yes	32.54	1909.53
leaves, phloem, aphids	D-Fructose, 1,3,4,5,6-pentakis-O-(trimethylsilyl)-, O-methyloxime	Fructose	yes	32.75	1920.73
aphids	D-(+)-Talose	Talose	no	33.08	1937.85
leaves, phloem, aphids	D-Glucose, 5TMS derivative	Glucose	yes	33.40	1954.85
leaves, phloem	L-Tyrosine, 3TMS derivative	Tyrosine, 3TMS	yes	33.49	1959.69
leaves, aphids	D-Mannitol, 6TMS derivative	Mannitol, 6TMS	yes	33.68	1969.45
aphids	D-Glucitol, 6TMS derivative	Glucitol, 6TMS	no	33.73	1971.93
phloem, aphids	Lactulose, octakis(trimethylsilyl) ether, methyloxime (isomer 1)	Lactulose	no	34.60	2018.17
aphids	unknown 4	unkown 4	no	34.85	2032.03
aphids	D-Gluconic acid, 6TMS derivative	Gluconic acid, 6TMS	no	35.05	2042.85
phloem, aphids	D-Ribose, 4TMS derivative	Ribose, 4TMS	no	35.52	2067.87
aphids	2-Hydroxymandelic acid, ethyl ester, di-TMS	2-Hydroxymandelic acid	no	35.92	2089.85
aphids	Dopamine, 4TMS derivative	Dopamine, 4TMS	no	36.09	2098.98

Sample Type	Compound name	Simplified compound name	Confirmation with derivatized standard	Retention time (minutes)	Kovats Index
aphids	Myo-Inositol, 6TMS derivative	Myo-Inositol, 6 TMS	no	36.60	2128.69
aphids	unknown 5	unknown 5	no	36.82	2141.57
aphids	unknown 6	unknown 6	no	37.00	2152.61
aphids	unknown 7	unknown 7	no	37.18	2162.91
aphids	D-Allose, oxime (isomer 1), 6TMS derivative	Allose, 6TMS	no	37.50	2182.05
aphids	D-Allose, pentakis(trimethylsilyl) ether, methyloxime (anti)	Allose	no	37.61	2188.30
leaves, aphids	D-(+)-Cellobiose, (isomer 1), 8TMS derivative	Cellobiose, 8TMS	no	37.88	2204.09
leaves	L-Rhamnose, 4TMS derivative	Rhamnose, 4TMS	no	38.41	2235.36
aphids	L-Tryptophan, 2TMS	Tryptophan, 2TMS	yes	38.52	2242.28
aphids	Stearic acid, TMS derivative	Stearic acid, TMS	no	38.65	2249.68
aphids	unknown 8	unknown 8	no	38.79	2258.19
aphids	unknown 9	unknown 9	no	39.39	2293.36
aphids	unknown 10	unknown 10	no	39.66	2309.94
aphids	unknown 11	unknown 11	no	40.21	2343.50
aphids	unknown 12	unknown 12	no	40.77	2377.45
phloem, aphids	D-Altro-2-Heptulose, 1,3,4,5,6-pentakis-O-(trimethylsilyl)-, O-methyloxime, 7-[bis(trimethylsilyl) phosphate	D-Altro-Heptulose	no	40.84	2355.63
phloem	unknown 13	unknown 13	no	41.13	2399.64
aphids	unknown 14	unknown 14	no	41.32	2411.96
aphids	unknown 15	unknown 15	no	41.48	2422.73
leaves, aphids	D-Glucuronic acid, 2,3,4,5-tetrakis-O-(trimethylsilyl)-, trimethylsilyl ester	Glucuronic acid	no	41.58	2429.19
aphids	Dulcitol, 6TMS derivative	Dulcitol, 6TMS	no	41.83	2445.52
aphids	Dulcitol, 6TMS derivative	Dulcitol, 6TMS	no	41.96	2454.63

Sample Type	Compound name	Simplified compound name	Confirmation with derivatized standard	Retention time (minutes)	Kovats Index
aphids	Uridine, 3TMS derivative	Uridine, 3TMS	no	42.33	2479.07
aphids	β-Gentiobiose, octakis(trimethylsilyl) ether	β-Gentiobiose	no	42.65	2500.20
aphids	D-(+)-Cellobiose, octakis(trimethylsilyl) ether, methyloxime (isomer 2)	Cellobiose	no	43.43	2551.23
aphids	unknown 16	unknown 16	no	43.54	2558.23
leaves, phloem, aphids	3-.alpha.-Mannobiose, octakis(trimethylsilyl) ether (isomer 1)	Mannobiose	no	43.70	2568.91
aphids	Inosine, 4TMS derivative	Inosine, 4TMS	no	44.32	2610.64
leaves, phloem, aphids	unknown 17	unknown 17	no	44.55	2626.56
aphids	unknown 18	unknown 18	no	44.78	2642.35
aphids	Adenosine, 4TMS derivative	Adenosine, 4TMS	no	45.19	2671.02
aphids	unknown 19	unknown 19	no	45.59	2698.82
leaves, phloem, aphids	Sucrose, 8TMS derivative	Sucrose, 8TMS	yes	45.77	2711.75
aphids	unknown 20	unknown 20	no	46.02	2729.17
aphids	3-α-Mannobiose	3-α-Mannobiose	no	46.56	2768.05
leaves	D-(+)-Turanose, octakis(trimethylsilyl) ether	Turanose	no	47.04	2802.49
aphids	D-(+)-Trehalose, octakis(trimethylsilyl) ether	Trehalose	no	47.24	2816.88
phloem	D-Lactose, octakis(trimethylsilyl) ether, methyloxime (isomer 1)	Lactose	no	47.35	2824.82
aphids	2-.alpha.-Mannobiose, octakis(trimethylsilyl) ether, methyloxime (isomer 2)	alpha-Mannobiose	no	47.83	2859.58

Sample Type	Compound name	Simplified compound name	Confirmation with derivatized standard	Retention time (minutes)	Kovats Index
aphids	2-.alpha.-Mannobiose, octakis(trimethylsilyl) ether, methyloxime (isomer 1)	alpha-Mannobiose	no	48.01	2872.82
leaves	Aucubin, hexakis(trimethylsilyl) ether	Aucubin	no	48.15	2883.33
aphids	Maltose, octakis(trimethylsilyl) ether, methyloxime (isomer 2)	Maltose	no	48.43	2903.54
aphids	unknown 21	unknown 21	no	48.75	2927.71
aphids	unknown 22	unknown 22	no	49.11	2955.67
aphids	Lactose, 8TMS derivative	Lactose, 8TMS	no	49.36	2974.15
aphids	D-(+)-Turanose, octakis(trimethylsilyl) ether	Turanose	no	50.45	not calculated
aphids	unknown 23	unknown 23	no	50.93	not calculated
aphids	unknown 24	unknown 24	no	51.20	not calculated
aphids	unknown 25	unknown 25	no	51.35	not calculated
aphids	unknown 26	unknown 26	no	51.59	not calculated
aphids	unknown 27	unknown 27	no	51.96	not calculated
aphids	unknown 28	unknown 28	no	55.38	not calculated
aphids	unknown 29	unknown 29	no	56.25	not calculated
aphids	unknown 30	unknown 30	no	57.57	not calculated
